# Prioritized experience replays on a hippocampal predictive map for learning

**DOI:** 10.1073/pnas.2011266118

**Published:** 2020-12-28

**Authors:** Hideyoshi Igata, Yuji Ikegaya, Takuya Sasaki

**Affiliations:** ^a^Graduate School of Pharmaceutical Sciences, University of Tokyo, Bunkyo-ku, 113-0033 Tokyo, Japan;; ^b^Center for Information and Neural Networks, National Institute of Information and Communications Technology, Suita City, 565-0871 Osaka, Japan;; ^c^Institute for AI and Beyond, University of Tokyo, Bunkyo-ku, 113-0033 Tokyo, Japan;; ^d^Precursory Research for Embryonic Science and Technology, Japan Science and Technology Agency, Saitama, 332-0012 Kawaguchi, Japan

**Keywords:** hippocampus, replay, place cell, learning, ripple

## Abstract

Hippocampal cells are central to spatial and predictive representations, and experience replays by place cells are crucial for learning and memory. Nonetheless, how hippocampal replay patterns dynamically change during the learning process remains to be elucidated. Here, we designed a spatial task in which rats learned a new behavioral trajectory for reward. We found that as rats updated their behavioral strategies for a novel salient location, hippocampal cell ensembles increased theta-sequences and sharp wave ripple-associated synchronous spikes that preferentially replayed salient locations and reward-related contexts in reverse order. The directionality and contents of the replays progressively varied with learning, including an optimized path that had never been exploited by the animals, suggesting prioritized replays of significant experiences on a predictive map. Online feedback blockade of sharp wave ripples during a learning process inhibited stabilizing optimized behavior. These results implicate learning-associated experience replays that act to learn and reinforce specific behavioral strategies.

## Significance

The hippocampus is crucial for spatial learning but their neuronal mechanisms remain unknown. Here, we found that hippocampal cell ensembles preferentially replayed salient reward-related locations as rats learned a new behavioral trajectory for reward. The contents of these hippocampal replays progressively varied with learning. Especially during learning, hippocampal neurons even replayed an optimized path that had never been exploited by the animals. The learning-related hippocampal activity was necessary for the stabilization of spatial behavior. These results suggest prioritized replays of significant experiences on a predictive map by hippocampal neurons.

To adapt to continuous changes in the external environment, animals need to encode new salient episodes and update their behavioral policy. To achieve flexible spatial navigation in particular, hippocampal place cells are thought to provide fundamental neural spatial coding and constitute a cognitive map (1). Recently, the view of the cognitive map has been proposed to extend to a predictive map theory in which place cells potentially encode expectations about an animal’s future states (2). In addition to these map representations, hippocampal place cells that encode animals’ past or future trajectories are sequentially activated during sharp wave ripples (SWRs) within a short time window (∼100 ms) in a phenomenon known as “place cell replays” (3–5). The time window of such SWR-associated sequential firing is potentially appropriate to induce plastic changes in the hippocampal circuit. Consistent with this idea, a growing amount of evidence demonstrates that the frequency of SWR-associated place cell replays (or reactivation) is prominently increased during learning of new experiences (online replays) (6) and during rest/sleep states after learning of new experiences (offline replays) (7). In addition, selective disruption of waking SWRs during spatial learning has been demonstrated to reduce subsequent task performance (8) and impair the stabilization and refinement of place cell maps (9). Taken together, these results suggest an essential role of SWR-associated replays of an animal’s experiences in novel spatial learning.

Experience replays by place cells are also suggested to be a key neuronal basis for a reinforcement learning framework (10, 11). When agents encounter a prediction error, such as a change in reward, experience replays may be an efficient mechanism for the evaluation of their experienced action–outcome associations by providing a solution to the temporal credit assignment problem, which is helpful to update their behavioral policy to maximize future reward (12, 13). In line with this idea, empirical observations have demonstrated that the receipt of reward or novel experiences leads to increased rates of SWRs and coordinated reactivation of place cells (6, 14), and increased reward leads to increased reverse replays (4, 15).

Despite accumulating evidence and theory, the field still lacks key insights into how the contents of place cell replays change to (re)assign the values of salient locations as animals develop new navigation strategies in response to environmental changes. Theoretical works demonstrate that incorporating replay algorithms with prioritized memory access for learned salient locations (16), rather than random access from all stored memory, into a reinforcement learning architecture—a strategy termed “prioritized experience replays” (17)—improves integrative learning in artificial agents. Whether living neuronal networks adopt the same computations to enhance learning capability remains an open question. To address this issue, we designed a spatial learning task requiring rats to learn new spatial navigation within a recording period within an hour, and analyzed how hippocampal replays of salient locations associated with learning progressively varied with the animal’s learning processes.

## Results

### A Spatial Learning Task.

**Fig. 1.**
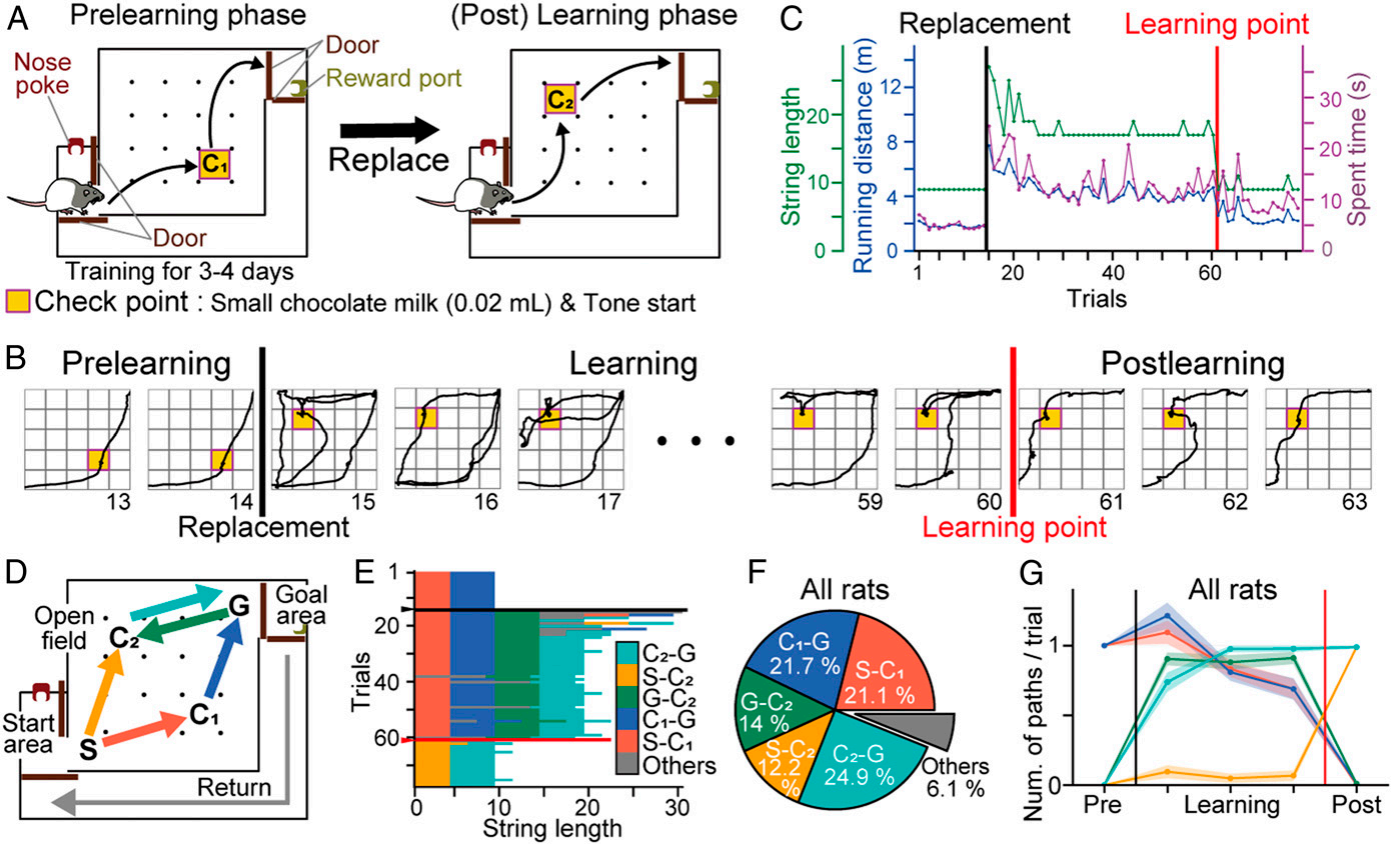
Behavioral performance in the spatial learning task. (*A*) Overview of the task. The rat first took a trajectory from the start to the goal area via C1, where a reward was placed (path S-C1-G; prelearning phase). The areas were separated by automatic doors. The reward point was then changed to C2, and the rat learned to take a new trajectory via C2 (learning phase). (*B*) Representative trajectories observed from a single rat (all trajectories are shown in *SI Appendix*, Fig. S2*A*). The black and red vertical lines indicate reward replacement and a learning point defined based on a learning curve, respectively. Trial numbers are indicated below the images. (*C*) Changes in running distance, duration, and string length for the rat. (*D*) Five paths analyzed. (*E*) Changes in string length for the rat. The black and red horizontal lines indicate the replacement and learning points, respectively. (*F*) Proportions of paths taken by all five rats. (*G*) Changes in the number of paths per trial. The colors correspond to the paths in *D*. The thin shaded regions indicate the SEM.

In this study, rats performed a spatial task in which they ran from a starting (S) area to checkpoint 1 (C_1_) (path S-C_1_), where they received a small (20 μL chocolate milk) reward, and then ran from C_1_ to a goal (G) area (path C_1_-G), where they received a large (200 μL chocolate milk) reward (Fig. 1*A* and *SI Appendix*, Fig. S1 *A*–*D*). On a recording day, after the animals were well trained on this task, the rats first performed the same task, termed the “prelearning phase.” After 11 to 14 trials, the reward was replaced to a new checkpoint (C_2_) (Fig. 1 *A*, *Right*), requiring the rats to learn new trajectories through trial and error. After the replacement, the rats first persisted in traversing path S-C_1_-G, the previous efficient trajectory; however, the reward was no longer presented. In an initial learning phase, the rats visited C_2_ via path G-C_2_ after taking path S-C_1_-G. As the learning phase proceeded, the rats gradually spent less time on path S-C_1_-G and instead settled on the most efficient trajectory, path S-C_2_-G (Fig. 1 *B*–*E*; for all rats, see *SI Appendix*, Fig. S2). For each rat, a learning point was determined based on a moving-average learning curve, and the periods before and after the learning point were termed the “learning phase” and the “postlearning phase,” respectively. The rats required 26 ± 9 trials (∼30 min) to progress from reward replacement to the learning point (*n* = 5 rats). In total, paths S-C_1_, C_1_-G, G-C_2_, S-C_2_, and C_2_-G accounted for 93.9% of all animal trajectories (Fig. 1 *F* and *G*). Compared with previous work, the concepts of this task were that 1) the consistency of task demands, irrespective of the locations of check points, enabled us to extract spatial and context-dependent neuronal codes (18, 19), and 2) an obvious learning point and the animals’ learning-dependent trajectories enabled us to analyze detailed hippocampal replay patterns associated with the rats’ internal evaluations of behavioral strategies.

### Spatial and Context-Dependent Spikes of Hippocampal Cells.

**Fig. 2.**
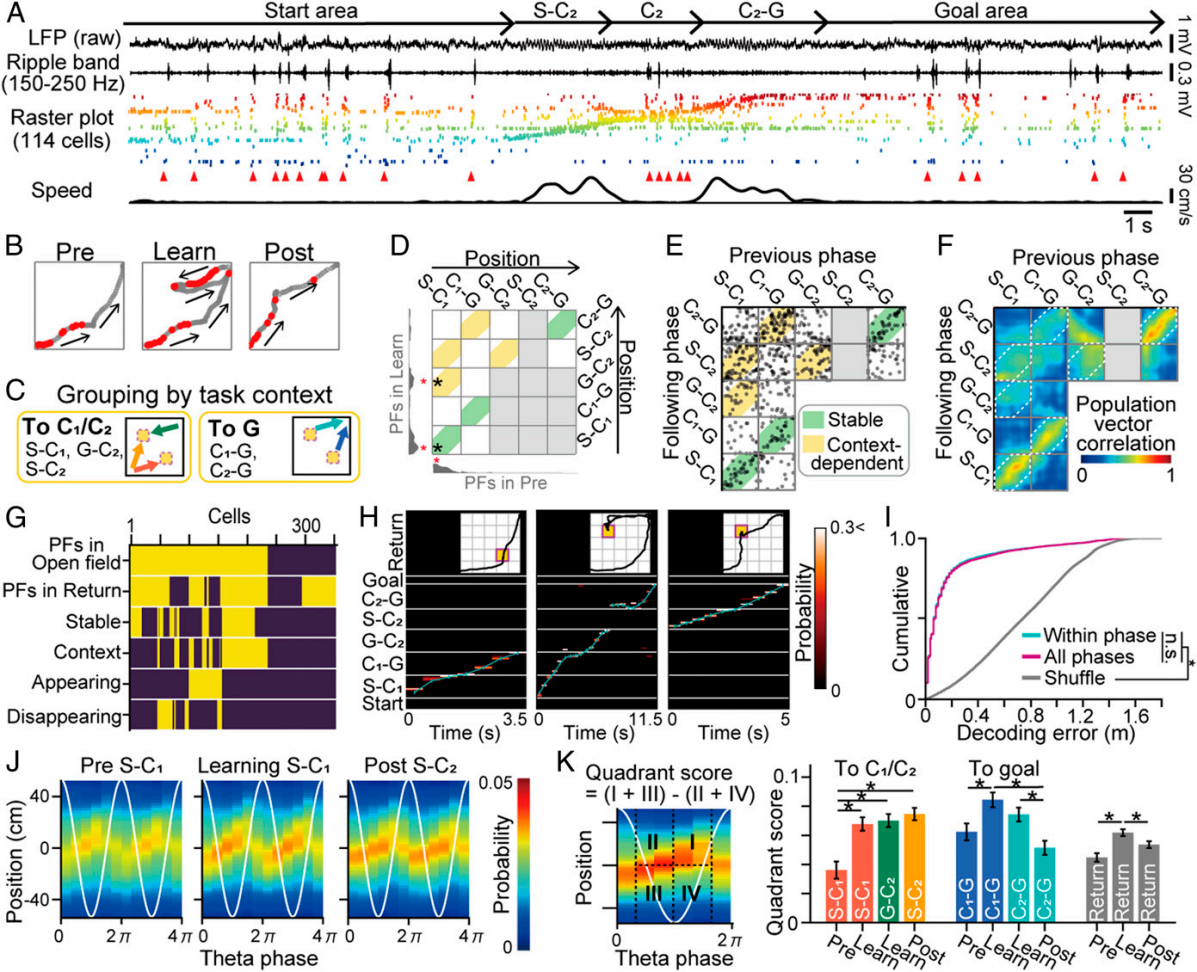
Spatial and context-dependent coding of hippocampal cells in the spatial learning task. (*A*) (top to bottom) Original local field potential (LFP), ripple band (150 to 250 Hz)-filtered LFP, raster plots showing the spike patterns of 114 neurons, arrowheads indicating synchronous events in the raster plot, and animal running speed in a trial during the postlearning phase. Place cells were ordered by their place-field locations. (*B*) Spatial firing of a place cell with both stable and context-dependent properties. Spike positions are represented as red dots superimposed on the trajectories (gray). This cell showed stable spatial representations on path S-C1 and specifically fired when the rat approached C1 or C2. (*C*) Definition of context-dependent firing. The trajectories shown on the left and right are common as the rats move toward the checkpoint (C1 or C2) and the goal, G, respectively. (*D*) Spatial firing rate distributions and the locations of place fields (shown as a red asterisk) of the place cell in *B* in the prelearning and learning phases are presented in the bottom and left, respectively. Joint place-field locations are plotted by black asterisks, representing the locations of all place-field pairs in different phases (green, stable; yellow, context-dependent). (*E*) All joint plots from all place cells. (*F*) Spatial correlation matrix of the population vector pairs at all location bins. (*G*) Summary of firing properties from all recorded cells (on, yellow; off, violet). (*H*) Bayesian decoding of rat trajectories from place cell spikes. Posterior probabilities of position estimates represented by a hot scale; position estimates overlaid with actual animal positions (cyan lines). (*I*) Errors of animal location estimates computed from datasets within each phase (cyan) or those averaged over all phases (magenta) by leave-one-out cross-validation: *P < 0.05, Mann–Whitney *U* test followed by Bonferroni correction. (*J*) Average posterior probabilities of rat positions while running on a path as described above: the *x* axis shows the phases of two theta-cycles (white lines), and the *y* axis shows the positions relative to the current animal’s location. (*K*) Comparison of quadrant scores of theta-sequences across paths and phases: *P < 0.05, Tukey’s test.

While the rats performed the spatial learning task, the spike patterns of 355 neurons in the dorsal hippocampal CA1 region were recorded with independently movable tetrodes from the five rats (Fig. 2*A* and *SI Appendix*, Fig. S3). Some place cells had multiple place fields distributed throughout the environment (Fig. 2 *B*–*E*; for all place fields, see *SI Appendix*, Figs. S3*F* and S4*G*). To examine learning-induced changes in the place field locations, all place fields from a single cell were visualized by joint plots (Fig. 2*D*; for all cells, see Fig, 2*E* and *SI Appendix*, Fig. S4 *A*–*C*). Based on the locations of place fields on the joint plots, we defined change characteristics for each place field. The majority of place fields were stable irrespective of learning (green regions in Fig. 2 *D* and *E*); however, a subset of new place fields emerged or disappeared in subsequent phases (*SI Appendix*, Fig. S4*B*). In addition to these place fields, high-density plots were detected for path S-C_1_ vs. S-C_2_, path G-C_2_ vs. S-C_2_, path S-C_1_ vs. G-C_2_ (approaching check points), and path C_1_-G vs. C_2_-G (approaching the goal area) (yellow regions in Fig. 2 *D* and *E*). The same tendencies were confirmed by population vector correlation matrices (Fig. 2*F* and *SI Appendix*, Fig. S3 *C*–*E*). These place fields that commonly emerged during an identical behavioral step are consistent with the findings of start-related (or path integration-based) and goal-directed spatial coding, reported in previous studies (20–23), and here considered as context-dependent fields (Fig. 2*C*), indicating generalized memory maps are formed within the hippocampal circuit. The proportions of place cells with stable and context-dependent place fields were 47.3% (112 of 237) and 48.9% (116 of 237), respectively (Fig. 2*G* and *SI Appendix*, Fig. S4*E*). Even when all these cell types, varying depending on different learning phases, were included in the analysis, Bayesian decoding could still accurately reconstruct the rat’s actual positions, even in similar contexts, from the position tuning curves of the cells at population levels (Fig. 2 *H* and *I* and *SI Appendix*, Fig. S3*G*) (*P* < 0.05, Mann–Whitney *U* test versus shuffled data, *U* = 7317960132.5).

To examine the possibility of plastic changes in the hippocampal circuit, we analyzed whether learning of new behavioral trajectories was related to a theta-sequence in which spatial firing of the place cells was organized in sequences within a theta-cycle (Fig. 2*J*) (24), which is considered a neuronal substrate that encodes immediate future and past locations and induces synaptic plasticity for memory acquisition during novel experiences (25). To quantify theta-sequence, quadrant scores were computed, which represent the strength of the theta-sequences. In all paths in the field and the return path during the learning phase, quadrant scores were significantly higher than those during the prelearning phase (Fig. 2*K* and *SI Appendix*, Fig. S5) (*P* < 0.05, Tukey’s test; the significance between the novel path S-C_2_ and path S-C_1_ in the prelearning phase was similarly observed even when Rat 1 with the largest number of cells was excluded). These results mean that the strength of theta-sequences becomes higher not only on novel paths (paths S-C_2_-G and G-C_2_) but also on previous paths or familiar paths (e.g., path S-C1-G and the return path) when animals need to learn novel spatial behavior. In the postlearning phase, the quadrant scores in path S-C_2_ remained significantly higher (*P* < 0.05, Tukey’s test). The learning-induced stronger theta-sequences suggest increased cholinergic signals and plasticity in the hippocampal circuits during an entire learning phase.

### Learning-Dependent Synchronous Events of Hippocampal Cell Spikes.

**Fig. 3.**
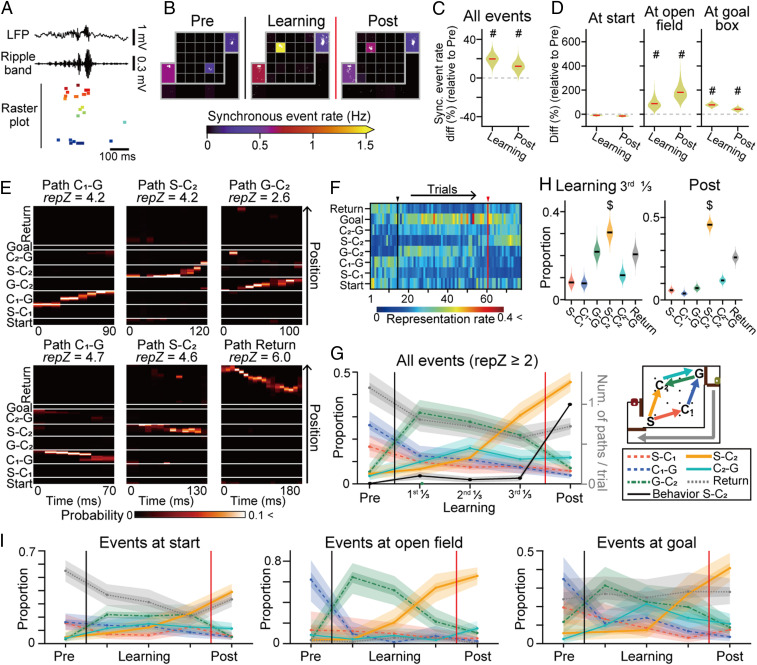
Prioritized representation of new episodes by learning-dependent synchronous events. (*A*) An SWR-associated synchronous event. (*B*) Pseudocolor maps of the average frequencies of synchronous events from all animals and trials, presented for a visualization purpose. The synchronous event locations are indicated by superimposed white dots. (*C*) Distributions of the percentage of changes in synchronous events during the learning and postlearning phases compared to the prelearning phase. A pound sign (#) indicates that 0 is not in the 95% credible interval computed from the posterior probability distribution by MCMC. (*D*) Same as *C*, but separately plotted for the individual areas. (*E*) Bayesian decoding of animal trajectories from single synchronous events (from Rat 1). Paths with the highest *z*-scored representation rates (*repZ*) are indicated above. The same datasets are magnified and the corresponding raster plots are presented in Fig. 4*A*. (*F*) Color-coded matrices of representation rates for each path by a single rat (the other four rats are shown in *SI Appendix*, Fig. S11*B*). The black and red vertical lines indicate the reward replacement and learning points, respectively. (*G*) Learning-related changes in the proportions of represented paths by synchronous events. The learning phase was evenly divided into three phases (first, second, and third learning phases). The thick and thin shaded areas indicate the 50% and 95% credible intervals, respectively. For comparison, the numbers of passes on path S-C_2_ per trial, presented in Fig. 1*G*, are superimposed by the black line. (*H*) The data in *G* were specifically analyzed for the last one-third of the learning and postlearning phases. A dollar sign ($) indicates an overlap of less than 5% with any other distribution. (*I*) Same as *G*, but for separately plotted individual areas.

We next analyzed how new spatial learning is related to SWR-associated synchronous spikes of neuronal populations within a short (∼100 ms) time window when animals stopped, termed “synchronous events” (Fig. 3*A* and *SI Appendix*, Fig. S6 *A*–*D*). In the prelearning phase, synchronous events were temporally sparse, with average frequencies below 0.4 Hz (Fig. 3*B* and *SI Appendix*, Fig. S7 *A*–*J*). The frequencies of synchronous events and SWRs in the learning and postlearning phases were quantified by the hierarchical Bayesian modeling with Markov chain Monte Carlo (MCMC) methods to take differences in the number of trials and events across individual rats into account (*SI Appendix*, Fig. S6*E*). We found that these events became noticeably higher in the open field and goal area compared with the prelearning phase (Fig. 3 *B*–*D* and *SI Appendix*, Fig. S7*J*) (the distributions computed by the hierarchical Bayesian modeling with MCMC methods exhibited overlaps of less than 5%). The numbers of synchronous events did not significantly differ across the learning subphases (*SI Appendix*, Fig. S7*B*). On the next day (day 2) after learning, such SWR increases were no longer observed, and the frequencies were comparable to those in the prelearning phase (*SI Appendix*, Fig. S8), confirming that novel learning was necessary to enrich synchronous events.

To examine the changes in neuronal ensemble patterns involved in synchronous events, correlation coefficients of vectorized population spikes were computed between synchronous events (*SI Appendix*, Fig. S9*A*). Event-to-event and trial-to-trial correlations changed substantially from learning (*SI Appendix*, Fig. S9 *B*–*F*), demonstrating that learning recruited new cell ensembles in synchronous events. To analyze the detailed behavioral episodes represented by these synchronous events, Bayesian decoding was applied to estimate the animal trajectories (Fig. 3*E*, *SI Appendix*, Fig. S10, and Movie S1), and the representation rates (*reprates*) for the individual paths were computed as their posterior probabilities (Fig. 3*F*, *SI Appendix*, Fig. S11, and Movie S2). The majority of synchronous events had a representation rate at a particular path segment that was prominently higher than those of the other segments (*SI Appendix*, Fig. S11 *F*–*L*), demonstrating that most of the synchronous events primarily represented one path segment. For each synchronous event, a represented path was defined as the path with the highest *reprate*. In the prelearning phase, the majority of the represented paths were paths S-C_1_, C_1_-G, and the return path, corresponding with the actual trajectories taken by the animals. During the learning phase, paths G-C_2_ and C_2_-G became the majority of represented paths (Fig. 3*G* and *SI Appendix*, Fig. S11*C*), corresponding to the animal’s trial-and-error running behavior along path G-C_2_-G (Fig. 1 *E*–*G*). Notably, during the last one-third of the learning phase—before the rats finally settled on path S-C_2_-G—the largest fraction of synchronous events changed to represent path S-C_2_ (Fig. 3 *G* and *H*) (overlaps of the distributions computed by the hierarchical Bayesian modeling with MCMC methods were less than 5%), and such dynamic changes in representation patterns were more apparent in the open field than in the start and goal areas (Fig. 3*I*). These results demonstrate that future efficient behavioral episodes were already represented by synchronous events even before the animals changed their behavioral strategy. The fact that it took several trials (16.4 ± 19.5 trials; mean ± SD) from the onset of increases in the representation of path S-C_2_ to the actual learning point (*SI Appendix*, Fig. S11*E*) suggests that hippocampal replay patterns do not necessarily lead to immediate changes in behavioral patterns. In addition, few observations of passes on path S-C_2_ in the last one-third of the learning phase (Figs. 1*G* and 3*G* and *SI Appendix*, Fig. S2*C*) suggest that rats’ internal simulations of efficient behavioral strategies, rather than actual behavioral experiences, primarily determine hippocampal replay patterns.

Another notable observation was that synchronous events more preferentially represented paths G-C_2_ and S-C_2_, where a reward (20 μL) newly emerged at the destination (C_2_), than path C_2_-G, where the a reward (200 μL) was continuously presented at the well-known destination (goal area), which indicates that novel reward-related action policy, rather than the amount of reward, determines the priorities of episodes to be represented by hippocampal synchronous events (14, 15). Taken together, these results suggest that hippocampal synchronous events prioritize representations of salient episodes in reference to internal prediction errors and that such representation patterns undergo dynamic changes as learning proceeds.

### Learning-Related Changes in the Directionality of Hippocampal Replays.

**Fig. 4.**
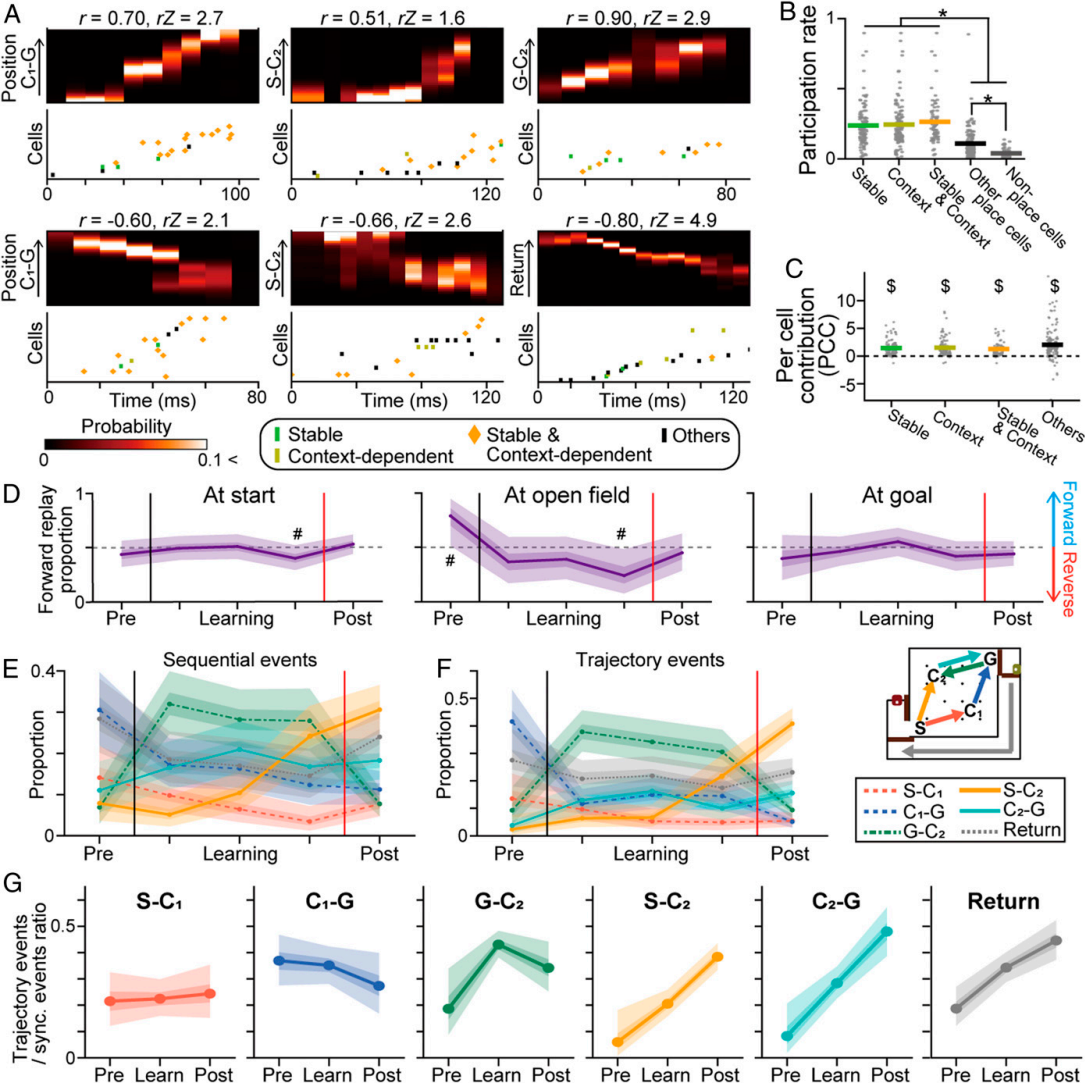
Representations by learning-dependent replay events. (*A*) Magnified views of the synchronous events shown in Fig. 3*E* and corresponding raster plots of the place cell spikes. The spikes are labeled based on cell type. (*B*) Participation rates of individual cells in synchronous events (*n* = 112, 116, 74, 154, and 47 cells). The gray dots represent each cell, and the colored lines represent the average: *P < 0.05, Tukey’s test. (*C*) PCC of individual cells to replay events: ^$^*P* < 0.05, one-sample *t* test versus 0. (*D*) The directionalities of replay events in individual areas. The proportions and statistical significance were computed at each time point. For a visualization purpose, these different time points were connected. The thick and thin shaded areas indicate the 50% and 95% credible intervals, respectively. A pound sign (#) indicates that 0.5 is not in the 95% credible interval. (*E* and *F*) Learning-related changes in the proportions of the represented paths by replay events with sequential and trajectory events. (*G*) The proportion of synchronous events assigned as trajectory events for each path. The thick and thin shaded areas indicate the 50% and 95% credible intervals, respectively.

To examine whether the learning-associated synchronous events corresponded with replay events consisting of temporally compressed sequential firing of hippocampal cells, the directionality and sequence strength of the spike trains for each synchronous event were assessed by computing a weighted correlation (*r*) and sequence score (*rZ*), respectively: Synchronous events where *r* ≥ 0.5 were considered sequential events that were classified into forward or reverse replay events (Fig. 4*A* and *SI Appendix*, Fig. S13 *A*–*C*). The participation rates of place cells with stable and context-dependent place fields in synchronous events were significantly higher than those of the other cells (Fig. 4*B* and *SI Appendix*, Fig. S12 *A*–*C*) (*P* < 0.05, Tukey’s test). In addition, these cell types contributed significantly to sequential events, as revealed by their significantly higher per cell contributions (PCCs) (Fig. 4*C* and *SI Appendix*, Fig. S12*D*) (*t*_104_ = 11.4, *t*_107_ = 9.8, *t*_68_ = 8.8, *t*_145_ = 9.1, *P* < 0.05, one-sample *t* test vs. 0). These results suggest that such stable and context-dependent representations cooperatively contribute to creating learning-dependent synchronous events by utilizing similar sets of hippocampal cells as a learned model for solving the task. In total, 345 of 2,586 (13.3%) and 390 of 2,586 (15.1%) synchronous events were classified as forward and reverse replay events, respectively (*SI Appendix*, Fig. S13*A*).

Consistent with previous observations (5, 26), sequential replay events in the open field were biased toward forward rather than reverse directions in the prelearning phase, confirming that forward representations before taking a path primarily emerge in learned situations (Fig. 4*D* and *SI Appendix*, Fig. S13*E*) (the overlaps between 0.5 and the distribution computed by the hierarchical Bayesian modeling with MCMC methods were less than 5%; the similar tendency of replay directionalities was observed but the significance was not detected in all comparisons when Rat 1 was excluded). On the other hand, this tendency was reversed after new learning was initiated; the reverse replays occupied significantly increased fractions in the latter learning phase, while the directionality of the replay events at the start and goal areas remained almost unchanged by learning. Especially, the reverse directionality was prominent in replays of path G-C_2_ (*SI Appendix*, Fig. S13*E*), a trajectory involved in the novel reward-related action policy, similar to the prominent increased representation (Fig. 3*G*). Overall, the sequential events showed learning-dependent changes in the proportions of represented paths similar to those in Fig. 3*G* (Fig. 4*E* and *SI Appendix*, Fig. S13*D*). In addition, we further validated these findings by computing trajectory events from the Bayesian decoding, which was another analytical method to detect hippocampal replays (5, 27). A similar tendency in learning-dependent changes in trajectory events were observed (Fig. 4 *F* and *G* and *SI Appendix*, Fig. S14*D*). These results demonstrate that hippocampal experience replays for the paths utilized in the new situation specifically developed with animal experience. Such learning-dependent changes in hippocampal replay patterns may be crucial for neuronal circuits to precisely reinforce new experiences that should be prioritized for learning.

### Requirement of Learning-Related Hippocampal SWRs in the Stabilization of Spatial Behavior.

**Fig. 5.**
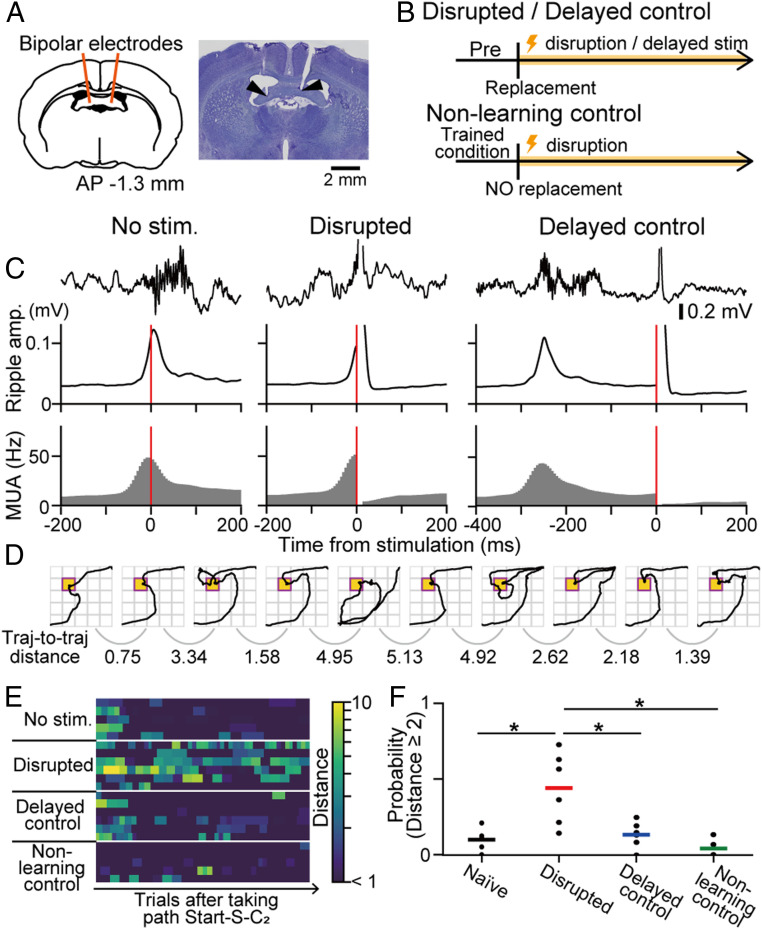
Impairment of learning by inhibition of SWRs. (*A*) Electrodes were bilaterally implanted in the vHC, and real-time closed-loop electrical stimulation was applied to disrupt hippocampal SWRs. (*B*) Schematic of the experiments. Timed or 250-ms delayed stimulation was applied upon SWR detection after reward relocation (disrupted or delayed control). In the nonlearning control group, the reward point was not relocated, but feedback stimulation was applied. (*C*) Original LFP traces, ripple power, and multiunit firing rates aligned to the time of stimulation in the unstimulated, disrupted, and delayed control groups. The red lines denote the time of stimulation. (*D*) Representative trajectories in a rat with disrupted SWRs. Trajectory-to-trajectory distances were computed between all pairs of successive trials. (*E*) Pseudocolor maps of the trajectory-to-trajectory distance for all individual rats after the animals first took the path start-S-C_2_. (*F*) The probability of trials with an observed distance ≥2 after the animals first took the path start-S-C_2_: *P < 0.05, Tukey’s test.

In order to test the causal role of SWR-associated synchronous events in learning performance, real-time disruption of SWRs was implemented by additionally implanting stimulation into the ventral hippocampal commissure (vHC); then, closed-loop feedback electrical stimulation with a single pulse (140 to 180 μA, 100 μs) was delivered upon the detection of hippocampal SWRs (Fig. 5 *A* and *B*). Similar to observations reported in earlier studies (8, 9), this manipulation could transiently eliminate SWR-associated neuronal synchronized events (Fig. 5*C*). Using this technique, we found that online disruption of ongoing SWRs after the reward replacement during a learning process resulted in unstable animal trajectories across trials (*SI Appendix*, Fig. S15 *B* and *C*), as demonstrated by a larger trajectory-to-trajectory distance (Fig. 5 *D* and *E*) and significant increases in the probability of behavioral changes across two successive trials after the animals first took the most efficient path (i.e., start-S-C_2_) (Fig. 5*F*) (*P* < 0.05, Tukey’s test). In contrast, stimulation delivered with a 250-ms delay relative to SWRs, applied as a delayed control experiment, and feedback stimulation in a nonlearning control animal group did not evoke such behavioral changes (Fig. 5 *E* and *F*) (*P* > 0.05, Tukey’s test). There were no significant differences in the number of trials to reach the learning point, the number of trials until the rats first took the path start-S-C_2_, and the number of trials from when rats first took the path start-S-C_2_ to the learning point (*SI Appendix*, Fig. S15 *D*–*F*) (*P* > 0.05 in all comparisons, Tukey’s honest significant difference post hoc test). The observation of SWR disruption-induced behavior deficit implies that learning-dependent SWRs and replay events support the evaluation and reinforcement of specific behavioral patterns during a learning process.

## Discussion

In this study, we designed a behavioral task in which rats could complete new spatial learning within a short recording time (up to 2 h), which allows us to analyze how hippocampal replay patterns undergo dynamic changes as the animals updated their navigational strategy through trial-and-error behavior. The main points of this study were: 1) That similar sets of hippocampal cells encoding specific places and behavioral contexts (e.g., approaching a specific point) are recruited in map representations across different learning phases; 2) that new spatial learning induces increases in hippocampal theta-sequences and sequential replays that preferentially represent salient (C_2_-related) experiences; 3) that the replays even represented an efficient path before the animals actually exploited the path; 4) that the contents and directionality (i.e., forward/reverse) of the replays progressively vary depending on learning phases; and 5) that such learning-related replays are necessary for stabilizing new optimized behavior.

The start point-related firing (e.g., on paths S-C_1_ and S-C_2_) might be explained by a firing property dependent on path integration of distance traveled from a specific location (20). On the other hand, the goal point-related firing (e.g., on paths C_1_-G and C_2_-G) might be partly explained by a firing property described as goal-directed (21, 22) and reward-predictive encoding (23). In addition, hippocampal cells encode information of time (28–30) and others (31, 32). Taken together, these observations suggest that hippocampal representations serve as a potential mechanism to map external environmental information into diverse spatial and nonspatial dimensions, as proposed by Aronov et al. (18), and hippocampal neurons are thus regarded as neuronal elements to represent generalized behavioral procedures and even process abstract factorized codes (19). These potential ideas over spatial representation could extend a hippocampal predictive map that has been primarily proposed for spatial information processing (2, 16) to a predictive model that can process more abstract information, which could further enrich predictive computations of task-state values and support model-based behavior (33).

Previous studies have shown that increases in hippocampal SWRs and replays are induced by novel experiences (6) and increased reward (14, 15, 34). Additionally, our observation that replay events more frequently encoded paths G-C_2_ and S-C_2_ (approaching the new checkpoint) than path C_2_-G (approaching a well-known goal) suggests that novel reward-related behavioral strategy, rather than the existence of reward, has the highest priority to be represented by replay events. Moreover, the observation of prioritized replays of the optimized path before updating the animal’s behavioral pattern suggests that internal simulations of future behavioral strategies, as well as actual behavioral experiences, can be incorporated into prioritized replays. At cellular levels, replay events more preferentially included spatially stable and context-dependent cells, compared with the other cells. These results suggest that the hippocampal circuit generates prioritized experience replays by flexibly recruiting these cell types depending on the demand of learning.

As the animals learned and developed new trajectory patterns, hippocampal cell ensembles prominently increased theta-sequences and SWR-associated replay events, both of which have been considered neurophysiological mechanisms to induce circuit plasticity (3, 9, 25, 35). Notably, compared with SWR-associated replays, the increases in theta-sequences were observed at broader areas, such as path S-C_1_-G and the return path, indicating that neuronal plasticity in the circuit occurs even when the animals take previous behavioral strategy that need to be abandoned or pass through an unchanged environment. The increases in theta-sequence reflects increased cholinergic signals throughout the learning phase, which potentially lead to effective synaptic plasticity when animals detect reward and reward-related dopaminergic signals are mobilized in the hippocampal circuit (35). Integration of these synapse-level mechanisms may enable the hippocampal predictive map to flexibly reorganize to properly represent varying task states when animals learn new behavioral policies in reference to their past episodes (2, 16, 36, 37).

In conclusion, the mammalian hippocampal circuit generates sequential replays with prioritizing salient experiences to effectively amplify and consolidate neuronal ensembles that encoded new experiences onto the circuit (*SI Appendix*, Fig. S16). Concordantly, recent studies of artificial intelligence have demonstrated that incorporating “prioritized experience replays” and episodic metalearning into deep neural networks could improve the efficiency of reinforcement learning in artificial agents (11, 12, 17, 36). Taken together, the evidence suggests that rehearsing salient external interactions by prioritized replays in a learned model is a common beneficial mechanism for both living brains and artificial agents to learn and reinforce specific behavioral strategies.

## Materials and Methods

The full methods can be found in *SI Appendix*

### Animals.

All experiments were performed with the approval of the experimental animal ethics committee at the University of Tokyo (approval no. P29-11) and according to the NIH *Guidelines for the Care and Use of Laboratory Animals* (38). A total of 17 male Long Evans rats (3- to 6-mo old) with preoperative weights of 400 to 500 g were used in this study. The animals were housed individually and maintained on a 12-h light/12-h dark schedule with lights off at 7:00 AM. All of the animal subjects were purchased from Japan SLC. The rats were reduced to 85% of their ad libitum weight by limiting daily feeding. Water was readily available.

### A Spatial Learning Task.

Each rat was trained to perform a spatial learning task. A rat initiated a trial by nose poking in the start area and obtained sucrose water during cue-sound presentation. Ten seconds after the onset of the sound presentation, the door between the start area and open field (door 1) was automatically opened, allowing the rat to enter the field. At the same time, 20 μL of chocolate milk reward was placed in the lattice fourth from left and second from bottom, termed checkpoint 1 (C1). The rat was trained to run from the first lattice after the start area to C1 (path S-C1), obtain the reward at C1 and then run from C1 to the last lattice before the goal area (path C1-G). When the rat entered C1, 5-kHz cue sounds were presented at 10 Hz for 0.3 s followed by continuous 10-kHz cue sounds until the rat reached the goal area. This sound cue helped a rat recognize that its current state was correct for obtaining chocolate milk in the goal area. When the rat reached the goal area after passing through C1, the door between the field and the goal area (door 2) was opened so that the rat could enter the goal area. In the goal area, the rat obtained 200 μL of chocolate milk reward. Twenty seconds after the onset of reward dispensation, the doors between the goal area and peripheral alleyway (door 3) and the between the peripheral alleyway and start area (door 4) were opened, allowing the rat to return to the start area through the alleyway to complete the trial. The next trial started when the rat again poked the reward port in the start area.

On a recording day, the rats first performed the same task with a reward placed on C1, termed the prelearning phase. After several trials, the rewarded check point was moved from C1 to the second lattice from the left and fourth from the bottom, termed checkpoint 2 (C2). In this phase, when the rat visited C2, 5-kHz cue were played at 10 Hz for 0.3 s followed by continuous 10-kHz cue sounds were presented until the rat reached the goal area. The chocolate milk reward volume and all of the other task conditions were similar to those in the prelearning phase. In this situation, the most efficient behavioral strategy was to run directly from S to C2 (path S-C2), take the reward placed on C2, and then run from C2 to G (path C2-G). After the reward replacement, the rats first exhibited trial-and-error behavior for several attempts to find an efficient trajectory, but they gradually learned to take the most efficient trajectory: Path S-C2-G. The detailed definition of a learning point is in *SI Appendix*.

### Surgical Procedures.

Five and 12 rats underwent surgery to implant recording electrodes only and a combination of recording and stimulating electrodes, respectively (22, 39, 40). Briefly, the rats were anesthetized with isoflurane gas (0.5 to 2.5%) and an electrode assembly consisting of 16 independently movable tetrodes was stereotaxically implanted above the right hippocampus (3.8-mm posterior and 2.8-mm lateral to bregma). For some animals, in addition, stainless bipolar electrodes were implanted at a depth of 3.7 mm at an angle of 6.9° into the right side or both sides of the vHC (1.3-mm posterior and 1.7-mm lateral to the bregma). Following surgery, each rat was housed individually in transparent Plexiglass with free access to water and food for at least 5 d and was then food-deprived until they reached 85% of their previous body weight. The electrode tips were slowly advanced by 25 to 100 μm per day for 11 to 24 d until spiking cells were encountered in the CA1 layer of the hippocampus.

### Electrophysiological Recording.

Electrophysiological data were sampled at 2 kHz and low-pass–filtered at 500 Hz. Unit activity was amplified and high-pass–filtered at 750 Hz. Spike waveforms above a trigger threshold (–50 μV) were time-stamped and recorded at 30 kHz for 1.6 ms.

### Closed-Loop Electrical Stimulation.

Upon the online detection of SWRs, closed-loop electrical stimulation was performed using an extension code implemented on the Cerebus recording system (Blackrock) and custom-created C code. At the time of SWR detection, an electrical pulse with a duration of 100 μs and an amplitude of 140 to 180 μA was applied to the vHC; the stimulation rate was limited to a maximum of 4 Hz. For delayed control stimulation, stimulation was applied with a latency of 250 ms after the onset of ripple detection so that the stimulation occurred outside the detected SWRs.

## Supplementary Material

Supplementary File

Supplementary File

Supplementary File

## Data Availability

Original datasets are available on the Mendeley Data repository at https://dx.doi.org/10.17632/4xk5w69yr5.1.
