# Supplementary material for: Prioritized experience replays on a hippocampal predictive map for learning
Source: Proc Natl Acad Sci U S A. 2020 Dec 28;118(1):e2011266118. doi: 10.1073/pnas.2011266118 (PMC7817193; doi:10.1073/pnas.2011266118)
Supplement: Supplementary File [file pnas.2011266118.sapp.pdf]

PNAS
www.pnas.org

Supplementary Information for

Prioritized experience replays on a hippocampal predictive map for learning

Hideyoshi Igata, Yuji Ikegaya, Takuya Sasaki

Hideyoshi Igata, Takuya Sasaki
Email: igata.hideyoshi@gmail.com and tsasaki@mol.f.u-tokyo.ac.jp

This PDF file includes:

Supplementary text
Figures S1 to S16
Legends for Movies S1 to S2
SI References

Other supplementary materials for this manuscript include the following:

Movies S1 to S2

www.pnas.org/cgi/doi/10.1073/pnas.2011266117

Supplementary Information Text

Materials and Methods

Behavioral apparatus for a spatial learning task

The overall size of the platform used for the spatial learning task was 1.2 m × 1.4 m, elevated 70 cm from the floor, with a wall height of 30 cm. This platform contained a square (1 m per side) open field, a 20 cm × 40 cm start area, a 20 cm × 30 cm goal area, and an L-shaped outer peripheral alleyway connecting the start and goal areas (return path) with a width of 20 cm (Fig. 1A and Supplementary Fig. 1B). The open field was evenly divided into 5 × 5 grids, and a black pole with a height of 10 cm and a diameter of 2 cm was placed at each grid point. The borders between the start area and the open field, the open field and the goal area, the goal area and the peripheral alleyway, and the peripheral alleyway and the goal were partitioned by automatic doors, termed door 1, door 2, door 3, and door 4, respectively (Fig. 1A and Supplementary Fig. 1B). All the doors were semiautomatically controlled. The other areas were partitioned by walls with a height of 22.5 cm. The floor, doors, and walls were all made of ABS resin. In the start area, a nose-poke reward port (3 cm in diameter) was attached to one side of the wall, and a white LED (5 × 5 mm) was attached 12 cm above the port. A speaker for sound presentation was placed outside the platform. In the goal area, an automatic reward-feeding port (7 × 6 cm) was attached to one side of the wall.

Trainings before surgery

All the behavioral experiments occurred in the dark phase with a light intensity of 1 lux. Training consisted of several steps (Supplementary Fig. 1C). On the first 2 days, each rat was habituated to the open field by allowing it to freely forage for randomly scattered chocolate milk for 10 min (Supplementary Fig. 1C, termed habituation).

After the habituation period, each rat was trained to perform voluntary nose poking in the start area for 2 days (Supplementary Fig. 1C, termed "nose-poke training"). Continuous nose poking for 1 s triggered 5-kHz sounds at 10 Hz for 0.3 s and a subsequent 10-kHz sound for 0.7 s. During the sound presentation period, the rat could obtain 25 μl of 30% sucrose eight times dispensed from the port by a syringe pump. Lighting of the white LED indicated that a reward was available at the port, and the LED was turned off when the rat could perform valid nose poking. After reward consumption, the rat had to leave the port for at least 2 s to initiate the next nose poking. When the intertrial intervals terminated after the rat left the port, the LED was again turned on to initiate the next trial.

Next, each rat was trained to run in the field (Supplementary Fig. 1C, termed "running training"). Similar to the nose-poke training, a rat initiated a trial by nose poking in the start area and obtained sucrose water during cue-sound presentation. Ten seconds after the onset of the sound presentation, the door between the start area and open field (door 1) was automatically opened, allowing the rat to enter the field. At the same time, 20 μl of chocolate milk reward was placed in the first lattice after the start area (S). When the rat entered lattice S, 5-kHz cue sounds at 10 Hz for 0.3 s followed by continuous 10-kHz cue sounds were presented until the rat reached the goal area. When the rat reached the last lattice before the goal area (lattice G), the door between the field and goal area (door 2) was opened so that the rat could enter the goal area. In the goal area, the rat received 200 μl of chocolate milk reward dispensed from the port by a syringe pump. Twenty seconds after the onset of entry into the goal area, the doors between the goal area and the peripheral alleyway (door 3) and between the peripheral alleyway and the start area (door 4) were opened, allowing the rat return to the start area through the alleyway to complete the trial (Supplementary Fig. 1D). During this running training, rats could follow any trajectory through the field from the start area to the goal area. On average, it took well-trained rats approximately 1 min to complete one trial. If a rat stayed in the field for more than 2 min

without opening door 2, door 2 was opened, irrespective of the rat's trajectory, so that the rat could enter the goal area. This training was repeated daily until the rat was able to complete more than 7 trials from the start to the goal area within 10 min. To achieve this performance criterion required between 5 and 7 days. After each rat met this criterion, it underwent surgery for electrode implantation.

To detect nose poking and reward consumption, infrared photoreflectors ware attached to the start and goal areas. To monitor the rat's moment-to-moment position, three red LEDs with a diameter of 5 mm were attached to the rat's back with a harness, and the positions of the LED signal were automatically tracked in real time at 24.5 Hz using a video camera attached to the ceiling. Depending on the instantaneous rat positions and sensor inputs, door openings and reward feedings were automatically regulated; these were digitized and timestamped by a laptop computer. Because all the apparatuses were controlled by a computer, it was not necessary for an experimenter to handle the rat after a task began.

Training for a spatial learning task after surgery

After recovery from surgery, the rats again underwent running training for 1–2 days (Supplementary Fig. 1C). After confirming that the rats had again reached the criterion for performance, as in the presurgery period, the rats were trained in the C_1 condition for 2–3 days (Supplementary Fig. 1C). Similar to the running training, a rat initiated a trial by nose poking and obtained sucrose water during cue-sound presentation. Ten seconds after the onset of the sound presentation, the door between the start area and open field (door 1) was opened automatically, allowing the rat to enter the field. At the same time, 20 μl of chocolate milk reward was placed in the lattice fourth from left and second from bottom, termed check point 1 (C_1). The rat was trained to run from the first lattice after the start area (S) to C_1 (path S-C_1), obtain the reward at C_1 and then run from C_1 to the last lattice before the goal area (G) (path C_1-G). When the rat entered $C_{1,}$ 5-kHz cue sounds were presented at 10 Hz for 0.3 s followed by continuous 10-kHz cue sounds until the rat reached the goal area. This sound cue helped a rat recognize that its current state was correct for obtaining chocolate milk in the goal area. When the rat reached G after passing through C_1, the door between the field and the goal area (door 2) was opened so that the rat could enter the goal area. In the goal area, the rat obtained 200 μl of chocolate milk reward. Twenty seconds after the onset of reward dispensation, the doors between the goal area and peripheral alleyway (door 3) and the between the peripheral alleyway and start area (door 4) were opened, allowing the rat to return to the start area through the alleyway to complete the trial. The next trial started when the rat again poked the reward port in the start area. At that point, all the doors were closed to initiate the next trial. In some cases, this training was performed with a recording headset and cable attached so that the animals became familiar with the recording condition.

To monitor the rat's moment-to-moment position, three red LEDs were attached to the electrode assembly, and the position of the LED signal was automatically tracked in real time at 24.5 Hz using a video camera attached to the ceiling. On all training and recording days, the rats were kept in a rest box (33 × 33 cm) outside the field for tens of minutes before and after performing the task.

A spatial learning task on a recording day

On a recording day, the rats first performed the same task with a reward placed on C_1, termed the prelearning phase. After several trials, the rewarded check point was moved from C_1 to the second lattice from the left and fourth from the bottom, termed check point 2 (C_2). In this phase, when the rat visited C_2, 5-kHz cue were played at 10 Hz for 0.3 s followed by continuous 10-kHz cue sounds were presented until the rat reached the goal area. The chocolate milk reward volume and all the other task conditions were similar to those in the prelearning phase. In this situation, the most efficient behavioral strategy was to run directly from S to C_2 (path S-C_2), take

the reward placed on C_2, and then run from C_2 to G (path C_2-G). After the reward replacement, the rats first exhibited trial-and-error behavior for several attempts to find an efficient trajectory, but they gradually learned to take the most efficient trajectory: path S-C_2-G. The detailed definition of a learning point is provided below.

Surgical procedures

Five and twelve rats underwent surgery to implant recording electrodes only and a combination of recording and stimulating electrodes, respectively. Briefly, the rats were anesthetized with isoflurane gas (0.5–2.5%), and a 2-cm midline incision was made from the area between the eyes to the cerebellum. For 5 rats, a craniotomy with a diameter of 0.9–1.6 mm was created above the right dorsal hippocampus (3.8 mm posterior and 2.8 mm lateral to bregma) using a high-speed drill, and the dura was surgically removed. Two stainless-steel screws were implanted in the bone above the prefrontal cortex to serve as ground electrodes. Using a 3D printer (Form 2, Formlabs) an electrode assembly consisting of 16 independently movable tetrodes was created and stereotaxically implanted above the craniotomy. The tips of the tetrode bundles were lowered to the cortical surface, and the electrodes were inserted 1.0 mm into the brain at the end of the surgery. The electrodes were constructed from 17-μm-wide polyimide-coated platinum-iridium (90/10%) wire (California Fine Wire California Fine Wire Co., Grover Beach, CA), and the electrode tips were plated with platinum to reduce their electrode impedances to 150–300 kΩ at 1 kHz. For 12 rats, an electrode assembly that consisted of 7 independently movable tetrodes was implanted using the same procedures as described above. In addition, the craniotomies (1.3 mm posterior and 1.7 mm lateral to the bregma) with a diameter of ~1 mm were created using a high-speed drill, and stainless bipolar electrodes were implanted at a depth of 3.7 mm at an angle of 6.9° into the right side or both sides of the ventral hippocampal commissure (vHC).

All the recording devices were secured to the skull using stainless-steel screws and dental cement (Provinice, Shofu Inc., Kyoto, Japan). Following surgery, each rat was housed individually in transparent Plexiglass with free access to water and food for at least 5 days and was then food-deprived until they reached 85% of their previous body weight.

Adjusting electrode depth

Each rat was connected to the recording equipment via a Cereplex M (Blackrock) digitally programmable amplifier, close to the rat's head. The output of the headstage was connected via a lightweight multiwire tether and a commutator to a Cerebus recording system (Blackrock), a data acquisition system. Electrode turning was performed while the rat was resting in a pot placed on a pedestal. The electrode tips were slowly advanced by 25–100 μm per day for 11–24 days until spiking cells were encountered in the CA1 layer of the hippocampus, which was identified on the basis of local field potential (LFP) signals and single-unit spike patterns. After the tetrodes were adjacent to the cell layer, as indicated by the presence of multiunit activity, the tetrodes were settled into the cell layer for stable recordings. Subsequently, recordings were conducted over a period of several days as described below.

Electrophysiological recording

Electrophysiological data were sampled at 2 kHz and low-pass filtered at 500 Hz. Unit activity was amplified and high-pass filtered at 750 Hz. Spike waveforms above a trigger threshold (–50 μV) were timestamped and recorded at 30 kHz for 1.6 ms.

Closed-loop electrical stimulation

Upon the online detection of SWRs, closed-loop electrical stimulation was performed using extension code implemented on the Cerebus recording system (Blackrock) and custom-created C code. A tetrode implanted into the hippocampus was chosen, and the envelope of its

bandpass (100–400 Hz)-filtered LFP signals at 30 kHz was estimated in real time as described previously (1). The smoothed estimate of the envelope (env_{est}) of filtered LFP signals was computed as follows:

$$env_{est}(t) = env_{est}(t-1) + gain(t-1) * (|v_{bp}| - env_{est}(t-1)),$$

where

$$gain(t) = \begin{cases} 0.013, & \text{if } |\mathrm{v_{bp}}| \le env_{est}(t-1) \\ mean(gain(t-600), gain(t-599), \ldots, gain(t-1), 0.08), & \text{if } |\mathrm{v_{bp}}| > env_{est}(t-1) \end{cases}$$

, and $|v_{bp}|$ denotes the absolute values of the filtered LFP signals. The length of the gain buffer was set to 600 (20 ms). Estimated values of smoothed mean ($mean_{est}$) and standard deviation (std_{est}) were then computed as follows:

$$mean_{est}(t) = mean_{est}(t-1) \times (N_{smooth} - 1)/N_{smooth} + |v_{bp}|/N_{smooth}$$

$$std_{est}(t) = \left(|v_{bp}| - mean_{est}(t-1)\right)/N_{smooth} + std_{est}(t-1),$$

where N_{smooth} was the number of samples for smoothing (typically, set to be 150,000 in 500 ms). SWRs were detected online when the animal's running speed was below 5 cm/s and when the envelope exceeded the detection threshold of 3–4 standard deviations above the estimated mean computed from LFP signals during periods in the rest box. At the time of SWR detection, an electrical pulse with a duration of 100 μs and an amplitude of 140–180 μA was applied to the vHC; the stimulation rate was limited to a maximum of 4 Hz. For delayed control stimulation, stimulation was applied with a latency of 250 ms after the onset of ripple detection so that the stimulation occurred outside the detected SWRs.

Histological analysis to confirm tetrode locations

After the experiments, the rats received an overdose of urethane and were intracardially perfused with 4% paraformaldehyde in PBS and decapitated. To aid in the reconstruction of the electrode tracks, the electrodes were not withdrawn from the brains until more than 3–4 hours after perfusion. After dissection, the brains were fixed overnight in 4% paraformaldehyde (PFA) and then equilibrated with a sequence of 20% sucrose and 30% sucrose in PBS. Frozen coronal slices (50 μm) were cut using a microtome (Sliding Microtome, SM2010 R, Leica Biosystems, Wetzlar, Germany), and serial sections were mounted and processed for cresyl violet staining. To perform cresyl violet staining, the slices were rinsed in water, counterstained with cresyl violet, and coverslipped with hydrophobic mounting medium (PARAmount-D, Falma, Tokyo, Japan). The positions of all the tetrodes were confirmed by identifying the corresponding electrode tracks in histological tissue with an optical microscope (All-in-One Fluorescence Microscope BZ-X710, Keyence Corporation, Osaka, Japan).

Spike sorting of hippocampal neurons

Spike sorting was performed offline using the graphical cluster-cutting software MClust (2). Rest recordings before and after the behavioral paradigms were included in the analysis to assure recording stability throughout the experiment and to identify hippocampal cells that were silent during the running behavior. Clustering was performed manually in two-dimensional projections of the multidimensional parameter space (i.e., comparisons between waveform amplitudes, the peak-to-trough amplitude differences, waveform energies, and the principal components of waveforms, each measured on the four channels of each tetrode). Autocorrelation and cross-correlation functions were used as additional separation criteria. Refractory periods of spikes were considered to increase confidence in the successful isolation of cells. Clustering

quality was measured by computing the L_{ratio} and isolation distance (3). A cluster was considered to be a cell when the L_{ratio} was less than 0.40. Overall, 114, 72, 68, 79, and 22 cells were recorded from rat 1, 2, 3, 4, and 5, respectively.

Analysis of animal trajectories and definition of a learning point

To analyze animal trajectory patterns, the open field was evenly divided into a 5 × 5 lattice and each grid cell was labeled with character strings "a-y" (Supplementary Fig. 1E). Five paths connecting specific pairs of four lattices (u (S), e (G), s (C_1), and g (C_2)), S-C_1, C_1-G, G-C_2, C_2-G, and S-C_2, were analyzed. The minimum string length of all these paths was 5. An animal's trajectory was divided into trajectory segments connecting two of the four specific lattices, and each trajectory segment was classified into one of the five paths if the length of the trajectory segment was less than 8 (an example shown in Supplementary Fig. 1G; red, S-C_1; blue, C_1-G; green G-C_2; cyan, C_2-G; orange, S-C_2). Trajectory segments that did not meet this criterion were classified as "other". The majority of trajectory segments were classified into paths S-C_1, C_1-G, G-C_2, S-C_2, and C_2-G (for more detail, see Fig. 1E and 1F). In all the following analyses, these five paths were selectively analyzed unless otherwise specified. For the process of linearizing the rat trajectories on individual paths, see Supplementary Fig. 1I.

For each rat, a learning point (between the learning and postlearning phases) was computed by depicting a learning curve with a moving average window of 5 trials (Supplementary Fig. 2B and 2D). In the learning phase, the optimized trajectory was defined as path S-C_2-G with a total string length less than 12. In a learning curve, a learning point was defined as one trial before the point where the optimized trajectory percentage first exceeded 50%. If that trial did not include the optimized trajectory, the learning point was defined as the next trial.

Place field characteristics

For each cell, an average spatial firing-rate distribution at an animal's running speed of more than 5 cm/s was computed along the linearized trajectories (Supplementary Fig. 1I) with a bin size of 2 cm; these data were then smoothed by a Gaussian filter (σ = 5 cm). For each distribution, a null distribution was constructed using an identical procedure, in which the spike locations were randomized while the total number of spikes was preserved. For one original distribution, 1,000 null distributions were created. The null distributions were created separately for the open field and the return path. The candidate position bins for the place fields were detected where the original firing rates exceeded the 99th percentile of those in the 1,000 null distributions. Place fields were defined as candidate position bins when more than five candidate position bins with firing rates greater than 1 Hz were detected consecutively as described previously (4). For each place field, the place field center (termed, place field location) was defined as the position with the maximum spiking rate. The place field detection was performed separately for each area and path (start area, S-C_1, C_1-G, G-C_2, S-C_2, C_2-G, goal area, and return). Neurons with at least one place field in the open field or return path in at least one of the three phases were classified as "place cells".

The characteristics of each place cell's place fields on the five paths (S-C_1, C_1-G, G-C_2, S-C_2, and C_2-G) were defined by depicting joint plots of place field locations between all phase pairs: prelearning versus learning phases, learning versus postlearning phases, and prelearning versus postlearning phases (an example cell is shown in Fig. 2D and all cells are shown in Fig. 2E and Supplementary Fig. 4). Place fields that emerged or disappeared in a later phase were classified as "appearing" or "disappearing", respectively. A place field that emerged at the same location with an interfield interval of less than 20 cm across two phases was classified as "stable". Place fields that commonly emerged when an animal approached or left specific lattices (S, C_1/C_2, and G) were classified as "context-dependent". A place field that emerged at the same location irrespective of the running direction on paths C_2-G and G-C_2 was classified as

“bidirectional”. Place cells with these place field characteristics are summarized in Fig. 2G and Supplementary Fig. 4D. According to this definition, multiple place field characteristics were defined from a single place cell, classifying place cells into “Stable”, “Context-dependent”, and “Stable & Context-dependent” cells. Cells that did not include any of these place field characteristics were classified into “Others” type cells, which included cells that had place fields in the start area, goal area, and return path, or appearing and disappearing place fields (Supplementary Fig. 4F).

Spatial correlation of place-cell population vectors

In each place cell, a spatial firing-rate distribution in each phase was normalized so that the maximum firing rate was 1, as shown in Supplementary Fig. 3A. In each phase, a population vector with a location bin size of approximately 2 cm was constructed from the normalized firing-rate distributions of all the recorded place cells. Correlation coefficients for all pairs of population vectors were computed to construct a population vector correlation matrix (Supplementary Fig. 3C and 3D) as follows:

$$\text{Correlation} = \frac{\boldsymbol{PV_1} \cdot \boldsymbol{PV_2}}{|\boldsymbol{PV_1}||\boldsymbol{PV_2}|},$$

where $\boldsymbol{PV}$ is a population vector in each phase.

Bayesian decoding of animal locations

To reduce memory requirements, uniform prior Bayesian decoding was applied to estimate the animals' positions from the spike trains. The spatial firing-rate distributions of individual place cells were used as position-tuning curves. As described previously (5), assuming Poisson firing statistics and a uniform prior over position, the posterior probability of the animal’s location (loc) in a time window (τ) including neuronal spike patterns (s) was computed as follows:

$$Pr(loc|s) = \cup / \textstyle\sum_{j=1}^{L} \cup$$

where

$$\cup = \left(\textstyle\prod_{i=1}^{N} f_i(loc)^{n_i}\right) \exp\left(-\tau \textstyle\sum_{i=1}^{N} f_i(loc)\right),$$

$f_i(loc)$ is the position tuning curve of the i-th neuron, and N and L are the total numbers of neurons and total location bins, respectively. The time window τ was set to 490 ms (12 video frames), 20 ms, and 21 ms (1/6 theta cycles) to estimate the animal’s positions at timescales of behavior (Fig. 1), synchronous events (Fig. 3E and 4A), and theta cycles (Fig. 2J), respectively. For visualization purposes and trajectory event detection, decoding for synchronous events was conducted with a 10-ms sliding window (Fig. 3E).

Fig. 2I shows a decoding accuracy comparison between the original position tuning curves and those from the surrogate position tuning curves in which the spatial selectivity of place cells was randomized. To create the surrogate position tuning curves, individual firing rates in the spatial firing-rate distributions were randomly shifted across all location bins for each cell before applying the Gaussian filter; then, the shuffled spatial firing-rate distributions were smoothed with a Gaussian filter (σ = 5 cm).

Theta sequence

LFP signals at a running speed of more than 5 cm/s were bandpass-filtered at the theta (6–10 Hz) band, and the peak and trough in each theta cycle were detected. Each theta cycle was evenly divided into 6 bins with a time window of ~21 ms, and Bayesian decoding of the animal’s positions relative to the current locations was performed in this time window as described above. The data obtained when the animals were located within 15 cm of the start and end points in each path were excluded from this analysis. The decoded probabilities (80 cm around the animal’s location and 2⁄3 of the theta cycle around the center of theta cycles) were divided into four

quadrants, and a quadrant score was computed as the difference in the average decoded probabilities between the second and fourth quadrants and the first and third quadrants (Fig. 2K), as described previously (6).

Detection of synchronous event

For each rat, the instantaneous spike rates (bin = 1 ms) were averaged over all the recorded neurons and smoothed with a Gaussian filter (σ = 15 ms). In addition, the mean and standard deviation (SD) of instantaneous spike rates during stop periods with a running speed of less than 5 cm/s were computed from all the recorded neurons, termed the $mean_{base\ rate}$ and $SD_{base\ rate}$, respectively (Supplementary Fig. 6C, left). Using these baseline variables, the instantaneous spike rates were z-scored as shown in the bottom trace in Supplementary Fig. 6A and Supplementary Fig. 6C. Candidate synchronous events were detected when the z-scored spike rates exceeded 2 and the number of active neurons exceeded 4. The onset and offset of candidate synchronous events were marked at the time points when the z-scored spike rates first exceeded and fell below 0, respectively. Candidate synchronous events in which the duration was less than 50 ms or more than 2000 ms were excluded from further analyses.

Next, the overall ripple power during candidate synchronous events was computed. In each tetrode, the LFP signals were bandpass filtered at 150–250 Hz, and the envelope of the filtered LFP traces was computed via the Hilbert transformation. Then, the envelope was smoothed with a Gaussian filter (σ = 4 ms). In addition, baseline mean and SD vectors of the smoothed envelopes during the stop periods were computed in each tetrode, termed $\boldsymbol{mean_{base\ power}}$ and $\boldsymbol{SD_{base\ power}}$, respectively. Based on these variables, the Mahalanobis distance was computed at each time point, termed overall ripple power.

Using the Gaussian mixture model (GMM), the distributions of the z-scored spike rate and z-scored overall ripple power in all candidate synchronous events were each divided into two distributions, as shown in Supplementary Fig. 6D. Synchronous events were detected when time points were included in the larger distributions of z-scored spike rate or z-scored overall ripple power (colored in magenta in Supplementary Fig. 6D).

Detection of SWRs

In a tetrode, LFP signals were bandpass filtered at 150–250 Hz, and the envelope of the filtered LFP traces was computed via the Hilbert transformation. The envelope was then smoothed with a Gaussian filter (σ = 4 ms), termed ripple power. In addition, the mean and SD of ripple power during stop periods with a running speed of less than 5 cm in the task periods were computed in each tetrode, termed $mean_{base\ power}$ and $SD_{base\ power}$, respectively. SWR events were detected when the envelope exceeded ($mean_{base\ power} + 3 \times SD_{base\ power}$). The onset and offset of SWR events were marked at the time points when the ripple power first exceeded and fell below $mean_{base\ power}$, respectively. SWR events with a duration less than 50 ms or more than 500 ms were excluded.

Similarity of synchronous events

To quantify the similarity of the synchronous events, each synchronous event was converted to an N-dimensional vector containing entries of the spike counts of individual neurons during the event, where N denotes the total number of neurons. Correlation coefficients between all possible vector pairs were calculated to construct an event-to-event correlation matrix (Supplementary Fig. 9A). A trial-to-trial correlation matrix was then constructed from the event-to-event matrix by calculating the average of all the correlation coefficients included in each trial pair except for the values on the diagonal line. Trials with fewer than 2 synchronous events were not analyzed. To evaluate the significance of the correlation coefficients in the trial-to-trial correlation matrix, the same analysis was applied to randomized data, in which the temporal order of the actual synchronous events in the original data was shuffled while preserving the total

numbers of synchronous events in the individual trials. This randomization process was repeated 1,000 times. At each point, a z-scored correlation coefficient of the real data was computed from the distribution of the 1,000 surrogate correlation coefficients (Supplementary Fig. 9B, bottom). A z-scored phase-to-phase correlation matrix was computed, similar to trial-to-trial correlations (Supplementary Fig. 9E and 9F).

Changes in the frequency of synchronous events and SWRs

Similar to the work of (7), hierarchical Bayesian modeling was applied to estimate the posterior probability of the number of synchronous events with the parameters estimated by Markov chain Monte Carlo (MCMC) methods (Supplementary Fig. 6E). We assumed that synchronous events emerge according to Poisson firing statistics and varied across individual animals, as follows:

$$\lambda = \exp(\beta_0 + \beta_1 \times learn + \beta_2 \times post + r_{rat} + \log(stay))$$

$$r_{rat} \sim \text{Normal}(0,\ s_{rat})$$

$$s_{rat} \sim \text{Uniform}(0,\ 10^4),$$

where λ is the expected value of the number of synchronous events, $stay$ is the duration (s) with a running speed of less than 5 cm, $learn$ and $post$ are dummy variables for the learning and postlearning phases, respectively. Here, r_{rat} reflects individual rat differences. Based on the estimated coefficients, the percentage of changes in the frequency of synchronous events for each trial in the learning and postlearning phases relative to those in the prelearning phase were computed as $100 \times [\exp(\beta_i) - 1]$, where *i* = 1 or 2, representing the learning or postlearning phases, respectively (Fig. 3C and 3D). Similarly, in Supplementary Fig. 7D, the percentage of changes in the number of synchronous events in each trial was computed with log(*stay*) = 0.

Similar to the synchronous events, the changes in SWR frequencies were quantified in Supplementary Fig. 7H, 7J, 8E, and 8G.

Representation rate

For each synchronous event, the posterior probability of the animal's location was computed by Bayesian decoding as described above (examples shown in Fig. 3E). The posterior probabilities within individual paths were averaged over the all location bins and summed over all the time bins. The summed posterior probabilities for the individual paths were then normalized so that the sum of the probabilities over all paths was 1, termed "representation rates". To depict the color-coded matrix in Fig. 3F, the representation rates of individual paths were averaged over each trial. For each synchronous event, a z-scored representation rate (*repZ*) was computed for the path segment with the highest representation rate as follows:

$$repZ = \frac{reprate - \text{mean}(reprate_{null})}{\text{s.d.}(reprate_{null})},$$

where *reprate* denotes a representation rate for the path segment and $reprate_{null}$ denotes the representation rates obtained from the decoding of the 1,000 surrogate synchronous events where the randomized position-tuning curves were created using the procedure described above.

Synchronous events representing individual path segments

Specifically, paths S-C_1, C_1-G, G-C_2, C_2-G, S-C_2, and the return path were analyzed. For each synchronous event, the representative paths were defined as the paths with the highest *reprate*. Hierarchical Bayesian modeling was applied to estimate the posterior probability of the proportions of represented paths with the parameters estimated by Markov chain Monte Carlo (MCMC) methods. We assumed that replays for the individual paths in each trial emerged according to a multinomial distribution as follows:

$$\boldsymbol{\Theta} \sim \text{Dirichlet}(\boldsymbol{\Theta}|\boldsymbol{\alpha})$$

$$\mathbf{data} \sim \text{Multi}(\mathbf{N}, \mathbf{\Theta}),$$

where **data** was a $T \times U$-dimensional vector matrix whose entries were the number of represented paths in each trial, T and U were the number of trials and paths (U = 6), respectively, $\boldsymbol{N}$ was a $T \times 1$-dimensional vector containing the total number of represented paths in each trial, and the parameters $\mathbf{\Theta}$ and $\boldsymbol{\alpha}$ were $U \times 1$-dimensional vectors for multinomial distribution and Dirichlet distribution, respectively. The initial vector $\boldsymbol{\alpha}$ was set to [1,1,1,1,1,1,1]. The parameter $\mathbf{\Theta}$ for the events where $repZ \geq 2$ was plotted as the proportions of represented paths in Fig. 3G, 3H, and 3I. The proportions of represented paths for all events are shown in Supplementary Fig. 11C and 11D.

Sequence scores of sequential events

Sequence scores were defined for individual synchronous events as described previously (4). For sequential events and trajectory events, the data from rat 1, 2, 3, and 4 were analyzed (with 68-114 cells). The path with the highest representation rate was analyzed. Replays for the start and goal areas were excluded from the analysis. For the represented path segments, $Pr(loc|s)$ constructed from a synchronous event was smoothed over time bins with a Gaussian filter (σ = 10 ms). In the filtered $Pr(loc|s)$, a maximum a posteriori probability (MAP) was computed as the largest posterior probability across all positions per time bin (8), and the time bins with MAPs greater than ($5 \times 1/n_{loc\ bin}$) were included for the analysis, where $n_{time\ bin}$ and $n_{loc\ bin}$ were the numbers of time and location bins in the synchronous event, respectively. If the number of time bins in which MAP exceeded the threshold was less than 3, all the time bins were analyzed. $Pr(loc|s)$ was normalized within the represented paths as follows:

$$normPr(loc|s) = \frac{Pr(loc|s)}{\sum_{i=1}^{n_{loc\ bin}} Pr\ (loc_i|s)}.$$

A sequence score *r(loc, time; normPr)* representing the weighted correlation between time and location was computed as follows:

$$r(loc, time, normPr) = \frac{cov(loc,time;normPr)}{\sqrt{cov(loc,loc;normPr)cov(time,time;normPr)}},$$

where

$$m(loc; normPr) = \frac{\sum_{i=1}^{n_{time\ bin}} \sum_{j=1}^{n_{loc\ bin}} normPr_{ij} loc_j}{\sum_{i=1}^{n_{time\ bin}} \sum_{j=1}^{n_{loc\ bin}} normPr_{ij}}$$

$$cov(loc; time; normPr) = \frac{\sum_{i=1}^{n_{time\ bin}} \sum_{j=1}^{n_{loc\ bin}} normPr_{ij}(loc_j - m(loc;normPr))(time_i - m(time;normPr)}{\sum_{i=1}^{n_{time\ bin}} \sum_{j=1}^{n_{loc\ bin}} normPr_{ij}}.$$

For each sequence score *r*, a z-scored sequence score (*rZ*) was computed as follows:

$$rZ = \frac{|r| - \text{mean}(|r_{null}|)}{\text{s.d.}(|r_{null}|)},$$

where r_{null} is the sequence score obtained from 1,000 surrogate synchronous events with randomized position tuning curves, which were randomly shifted within the represented path for each cell. Distributions of *r* and *rZ* values are presented in Supplementary Fig. 13A. Synchronous events with an $|r| \geq 0.5$ were considered sequential events, and the replay directions were determined by the signs of the correlations *r*, where positive and negative correlations of *r* represent forward and backward replay directions, respectively. Forward and reverse replay events were collectively termed sequential replay events.

Per cell contribution (PCC)

To quantify the contribution of each cell type (stable, context-dependent, and other place cells) to the sequence scores of synchronous events, PCCs were computed as described

previously (4). For each cell, 500 surrogate position-tuning curves were prepared by randomly shifting the position-tuning curve within the represented paths. The PCC of cell i to event e was computed as follows:

$$PCC_{e,i} = \left\{rZ_e - rZ_{e,i}(shuffle)\right\} \times Ncell_e,$$

where $rZ_{e,i}(shuffle)$ is the averaged rZ_e obtained from the shuffled tuning curves of cell i, and $Ncell_e$ is the number of activated place cells that encode the represented path in the event. For each cell, the PCCs were averaged over all events (Fig. 4C) or within each path (Supplementary Fig. 12).

The proportions of forward and reverse replays

Replay events for the start and goal areas were excluded from this analysis. Hierarchical Bayesian modeling was applied to estimate the posterior probability of the proportions of forward replays with the parameters estimated by Markov chain Monte Carlo (MCMC) methods. We assumed that the forward and backward replays in each trial emerged according to the binomial distribution as follows:

$$p \sim \mathrm{Beta}(p|\alpha, \beta)$$

$$\boldsymbol{data} \sim \mathrm{Binominal}(\boldsymbol{N}, p)$$

where **data** is a $T \times 1$-dimensional vector containing the number of forward replays in each trial, $\boldsymbol{N}$ is a $T \times 1$-dimensional vector containing the total number of replay events in each trial, the parameter p represents the binomial distribution, and the parameters α and β control the beta distribution and have initial values of 1. The parameter p was plotted as the proportion of forward replays in Fig. 4D and Supplementary Fig. 13E.

Trajectory events

A trajectory event was defined from a synchronous event, as previously described (5, 9). For each synchronous event, the posterior probability of the animal's location $Pr(loc|s)$ was computed by Bayesian decoding in which a 20-ms sliding window was moved every 10 ms as described above (*loc*: location, *s*: spikes). In the $Pr(loc|s)$, the maximum a posteriori probability (MAP) was computed for each path per time bin. Bins with MAPs greater than ($5 \times 1/n_{loc\ bin}$) were included in the following analyses, where $n_{loc\ bin}$ is the total number of all location bins.

A candidate trajectory sequence was detected when the distances between all pairs of neighboring locations giving the MAP (allowing a 1-gap time bin) were less than 40 cm within the represented paths. The candidate trajectory sequences were then extended to the neighboring paths using the same criteria, and the sequence with the maximum length was adopted as a trajectory sequence. Trajectory sequences covering more than 4 time bins (60 ms) and a cumulative distance of more than 20 cm were included in the further analyses.

To assess the significance of a trajectory sequence, p-values were computed to represent the probability of detecting trajectory events from the 1,000 surrogate synchronous events with randomized position tuning curves. These surrogate synchronous events were created using the same procedure as described above in the analysis for *repZ*. Trajectory events were defined from the trajectory sequences where $p < 0.05$.

Proportion of trajectory events

Hierarchical Bayesian modeling was applied to estimate the posterior probability of the proportion of trajectory events per synchronous event with the parameters estimated by Markov chain Monte Carlo (MCMC) methods. We assumed that the trajectory events in each trial emerged according to a binomial distribution as follows:

$$p \sim \mathrm{Beta}(p|\alpha, \beta)$$

$$\boldsymbol{data} \sim \text{Binominal}(\boldsymbol{N}, p),$$

where **data** is a $T \times 1$-dimensional vector containing the number of trajectory events in each trial, $\boldsymbol{N}$ is a $T \times 1$-dimensional vector whose entries are the total number of synchronous events in each trial, the parameter *p* represents the binomial distribution, and the parameters α and β control the Beta distribution and have initial values of 1. The parameter p was plotted as the proportion of trajectory events in Fig. 3G and Supplementary Fig. 14B and 14C.

Trajectory-to-trajectory distance

The convergence of behavioral trajectories across trials in the presence of closed-loop stimulation was quantified in Fig. 5. Specifically, we analyzed the trajectories after the rat first took path start-S-C_2 during the learning phase. A trajectory in the open field in each trial was converted to a character string (examples are shown in Supplementary Fig. 1F). A trajectory-to-trajectory distance was computed between two character strings in neighboring trials as a modified Levenshtein distance, with cost values considered as follows. A cost value for a string substitution was set to be (*distance*/1.13), where *distance* is the distance between the centers of two lattices involved in the substitution, and 1.13 is the distance (m) between lattices 'a' and 'y' representing the maximum lattice-to-lattice distance in the field (Supplementary Fig. 1E). The cost value for a deletion or insertion was 0.40/2, where 0.40 is the median of the normalized distance between all pairs of lattices.

When the trajectory-to-trajectory distance exceeded 2, the trial was considered to be a trial representing behavioral change. The percentage of trials with behavioral changes was computed for each rat, (Fig. 5F).

Statistical analysis

All the data were analyzed using MATLAB and Python. The data are presented as the mean ± standard error of the mean (SEM), dot plots with mean values, or box plots. Posterior probability distributions derived from the hierarchical Bayesian models with Markov chain Monte Carlo (MCMC) methods (10) are presented as violin plots or as lines showing the median values with 50% and 95% credible intervals.

Comparisons of one-sample data were analyzed using a one-sample *t*-test versus a constant value. Multiple group comparisons were performed by Tukey's tests (Fig. 2K, 4B, and 5F) or by Mann-Whitney U tests followed by Bonferroni corrections (Fig. 2I). The null hypothesis was rejected at the $p < 0.05$ level. Posterior probability distributions of the estimated parameters with hierarchical Bayesian modeling were considered to be significantly different when their probability of overlap was less than 0.05 (Fig. 3H and 4F).

DATA AND SOFTWARE AVAILABILITY

Original datasets are available at http://dx.doi.org/10.17632/4xk5w69yr5.1. MClust software is available from A.D. Redish at http://redishlab.neuroscience.umn.edu/MClust/MClust.html.

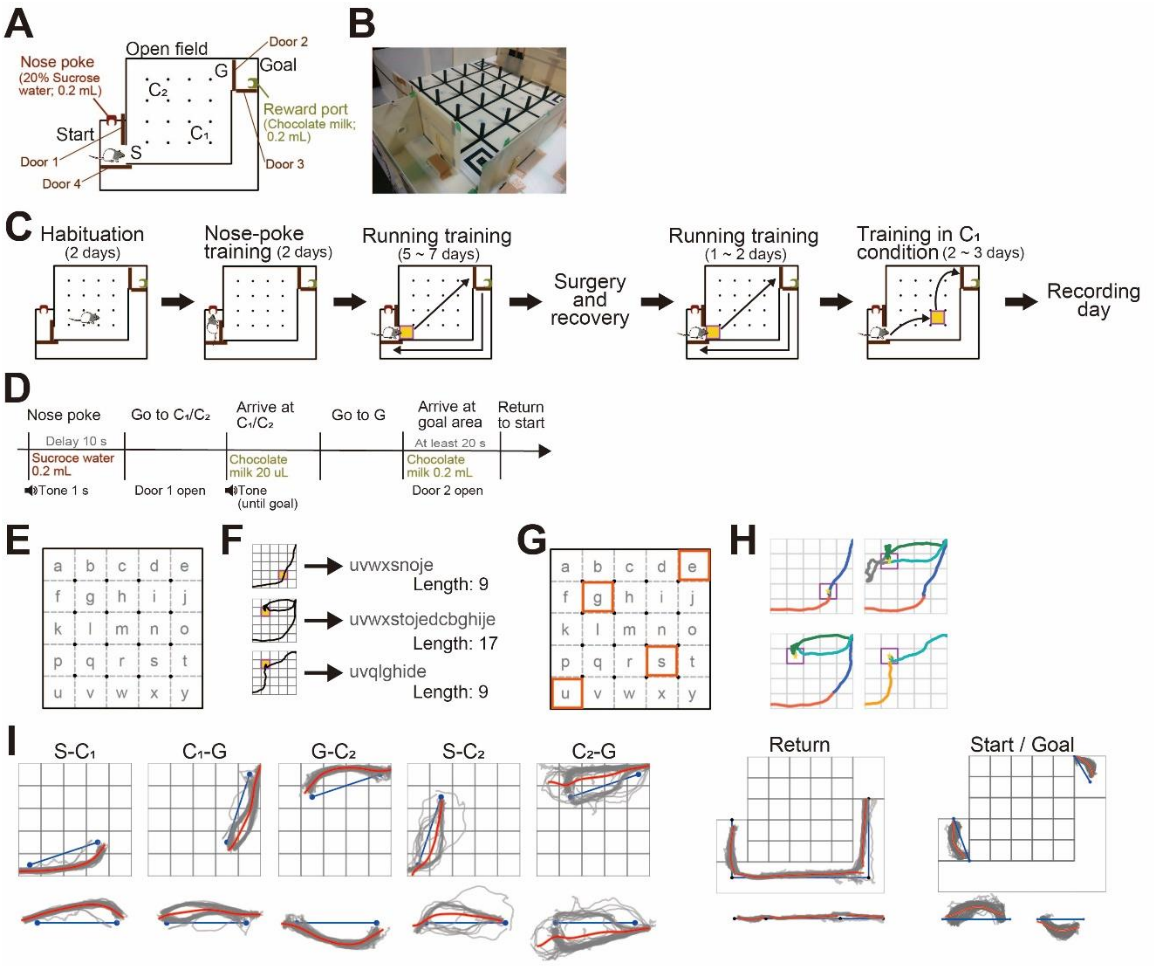

Fig. S1. Behavioral apparatus for the spatial learning task and analysis of animal trajectories. Related to Figure 1. (A) An overview of the area for the spatial learning task. "Nose poking" into the reward port in the start area for 1 s triggered the sound presentation and the dispensation of 25 × 8 **μ**l of 30% sucrose from the port. Next, door 1 was opened so that the rat could enter the open field and visit a checkpoint (C_1 or C_2) to obtain a 20 **μ**l chocolate milk reward (at one of the points C_1 or C_2). After reaching G, door 2 was opened so that the rat could enter the goal area. The reward port in the goal area then dispensed 200 **μ**l of chocolate milk reward. Finally, door 3 and door 4 were opened so that the rat could return to the start area via the return path. (B) A picture of the apparatus, viewed from the start area. (C) Timeline of training for the spatial learning task. (D) Time course of a single trial. (E) To analyze the trajectory patterns, the field was evenly divided into 5 × 5 lattices labeled with the letters “a–y”. The lattices u, e, s, and g correspond to S (after start), G (before goal), C_1, and C_2, respectively. (F) Three examples showing how the animals' trajectories were analyzed. A trajectory in the field was converted into a character string (target string, left), and the total number of characters in of each string was counted as the string length. (G) The paths connecting the pairs of four specific lattices (u(S), e(G), s(C_1), and g(C_2)) were analyzed. The majority of segments were classified into S-C_1, C_1-G, G-C_2, S-C_2, and C_2-G, which have a string length of 5. (H) Four representative rat trajectories are shown. A trajectory was divided into trajectory segments between the specific lattices, and each trajectory segment was classified into a path if the length of the segment was less than three plus

the length of the path. Path segments that did not meet this criterion were classified as "other". In the representative trajectories, each trajectory segment was colored based on the path (red, S-C_1; blue, C_1-G; green G-C_2; orange, G-C_2; cyan, C_2-G). (I) For each trajectory segment, a straight line connecting the centers of the two lattices at both ends of the path type (blue line) was depicted for projection. The blue line was aligned so that its start point was set as the origin and moved onto the x-axis by an affine transformation (bottom panels). Individual rat trajectory segments (gray traces) were projected onto the same axis. All the trajectories were divided into 2-cm bins on the line, and an averaged trajectory (red traces) was computed on the line.

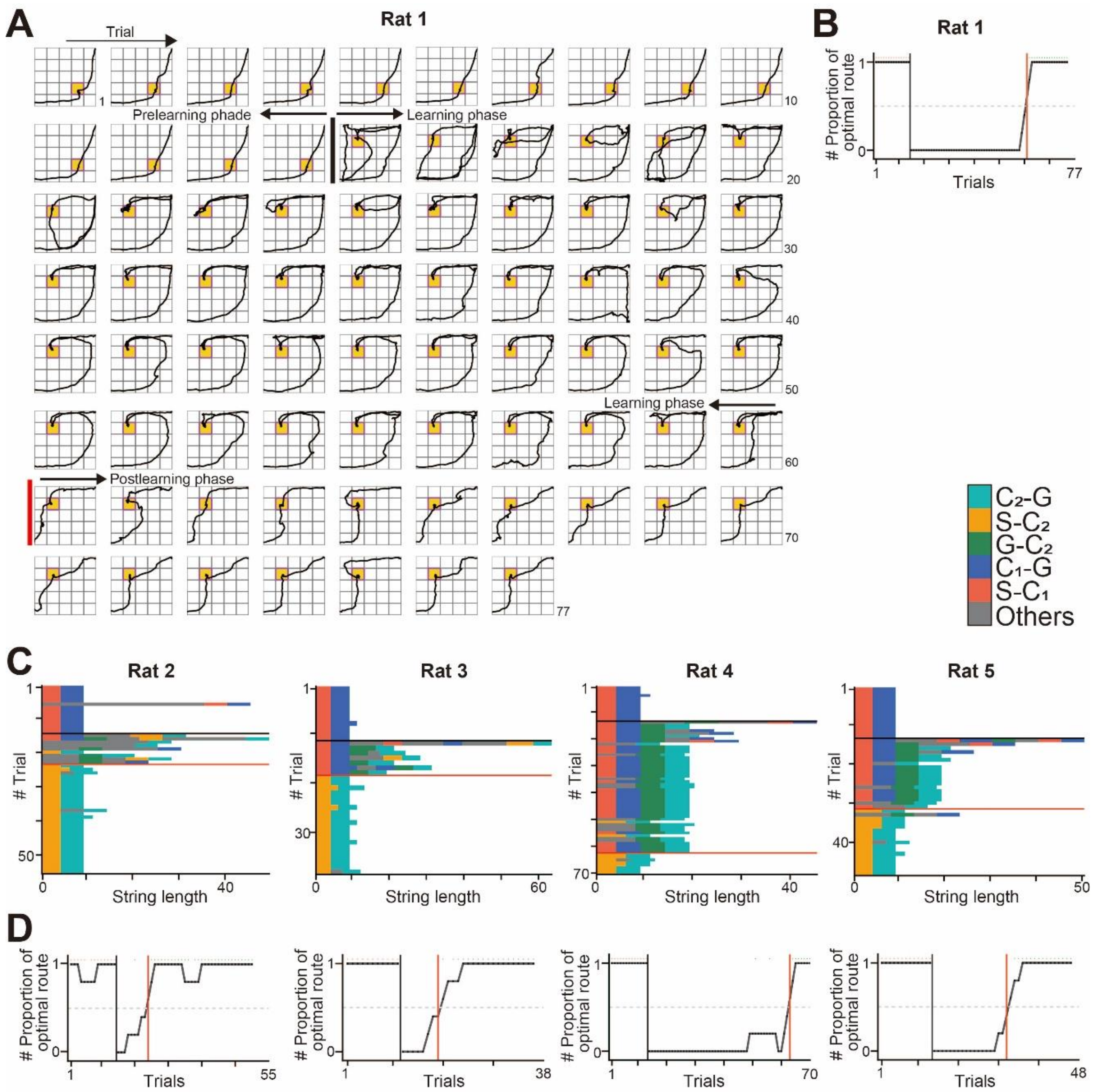

Fig. S2. Behavioral results of all rats. Related to Figure 1. (A) All the trajectories from a single rat (rat 1), corresponding with Fig. 1B. The black and red vertical lines indicate the reward replacement from C_1 to C_2 and the learning point, respectively. (B) A moving averaged learning curve, corresponding with A. (C) Changes in string length for each trial for the other four rats (rats 2–5). Data were plotted in the same way as in Fig. 1E. The black and red horizontal lines indicate the replacement and learning points, respectively. (D) Same as B but for the rats corresponding with C.

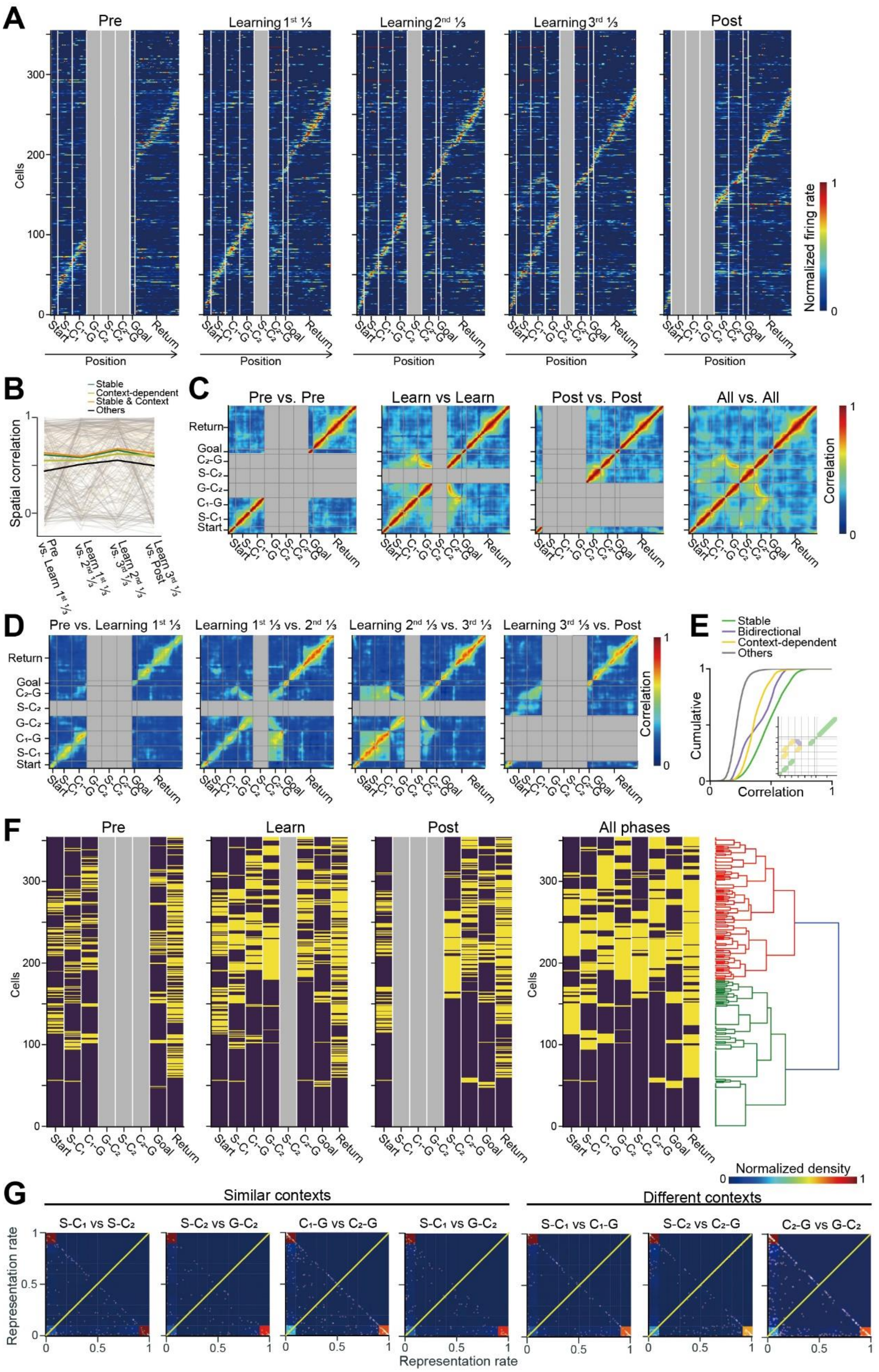

A
Pre
Learning 1st ⅓
Learning 2nd ⅓
Learning 3rd ⅓
Post
Cells
Normalized firing rate
Start
S-C1
C1-G
G-C2
S-C2
C2-G
Goal
Return
Position
B
Stable
Context-dependent
Stable & Context
Others
Spatial correlation
Pre vs. Learn 1st ⅓
Learn 1st ⅓ vs. 2nd ⅓
Learn 2nd ⅓ vs. 3rd ⅓
Learn 3rd ⅓ vs. Post
C
Pre vs. Pre
Learn vs Learn
Post vs. Post
All vs. All
Correlation
D
Pre vs. Learning 1st ⅓
Learning 1st ⅓ vs. 2nd ⅓
Learning 2nd ⅓ vs. 3rd ⅓
Learning 3rd ⅓ vs. Post
E
Stable
Bidirectional
Context-dependent
Others
Cumulative
Correlation
F
Pre
Learn
Post
All phases
Normalized density
G
Similar contexts
Different contexts
S-C1 vs S-C2
S-C2 vs G-C2
C1-G vs C2-G
S-C1 vs G-C2
S-C1 vs C1-G
S-C2 vs C2-G
C2-G vs G-C2
Representation rate

Fig. S3. Spatial firing patterns of all recorded hippocampal cells. Related to Figure 2. (A) Spatial firing-rate distributions of 355 recorded hippocampal neurons from 5 rats, ordered by the locations of their place field center computed from all sessions. Locations not visited by the rat did not visit are shown in gray. The data are separately shown for the prelearning phase, the three learning subphases, and the postlearning phase. In each cell, firing rates were normalized by the maximum firing rate observed throughout the task period. (B) Pearson's correlations of spatial firing-rate distributions between two neighboring (sub)phases were computed for each place cell. Thick lines represent average spatial correlations computed in each cell type (for cell-type classification, see Supplementary Figure 4), demonstrating that all cell types show prominent positive correlations of their spatial representations across the (sub)phases. Each thin line represents each cell. Some cells, especially for "Others" type cells showed sudden increases or decreases in their correlations due to appearing and disappearing place fields in some of the (sub)phases. (C) Spatial correlation matrices of population vector pairs at all location bins. The locations not visited by the rat are shown in gray. (D) To assess the changes in spatial firing patterns associated with learning a new rewarded location, spatial cross-correlation matrices were constructed from population vector pairs at all the location bins from neighboring (sub)phases. In all the matrices, the spatial correlations at similar locations were higher than the spatial correlations at different locations, demonstrating stable spatial representation by place-cell ensembles across the learning phases. Higher density correlations were detected at the comparison path C_2-G versus path G-C_2 in the learning phase, implying the presence of place fields independent of running directions, termed "bidirectional fields". Higher density correlations were also detected at S-C_1 versus S-C_2 and at C_1-G versus C_2-G, implying the presence of context-dependent fields relative to check points. (E) The cumulative distributions of correlation coefficients in specific regions (green, stable; yellow, context-dependent; purple, bidirectional) in the correlation matrices. The right bottom inset shows a schema depicting stable, context-dependent, and reverse fields (labeled by colors). (F) (Four left panels) Place-field locations of all recorded cells in individual phases (on, yellow; off, violet). The rightmost panel shows the hierarchical clustering of all recorded neurons based on their place-field locations. (G) Separation of decoded path segments. For each cell population activity during running in the open field (bin = 490 ms), Bayesian decoding was applied to compute representation rates (*reprates*) of individual path segments. The left four panels represent comparisons of two given path segments with similar contexts (approaching specific points) and the right three panels represent comparisons of two path segments that had no contextual relations. Each white dot indicates each cell population activity (bin = 490 ms), superimposed on a pseudocolor matrix of the normalized densities of all plots in the matrix. In all comparisons, the majority of dots was plotted on the regions with a *reprate* close to 1 in one path segment and a *reprate* close to 0 in the other segment, far from the diagonal lines (yellow lines), meaning that only one path segment was primarily represented by individual cell population activity, independently from the other path segments.

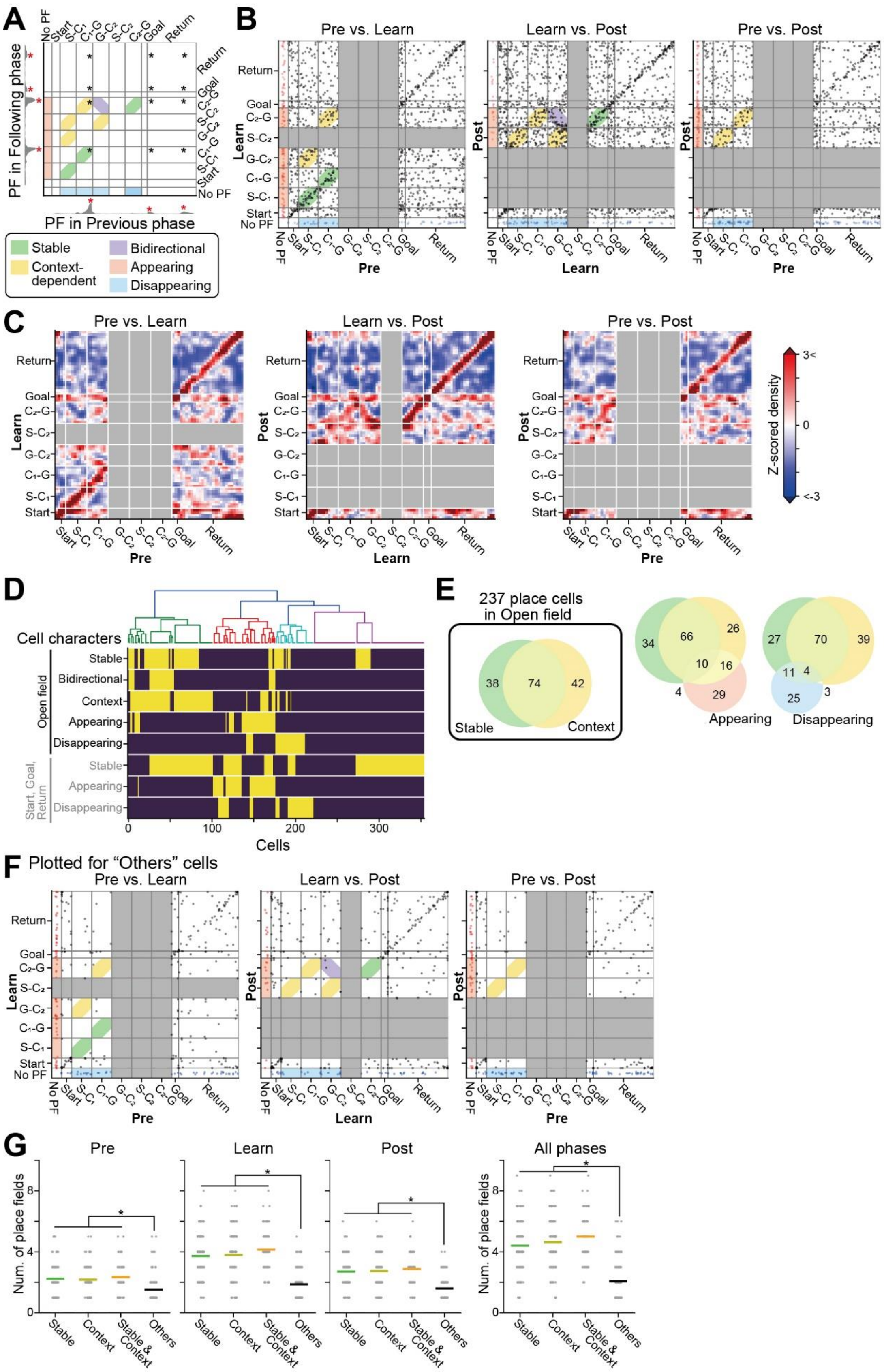
A
B
C
D
E
F
G
PF in Following phase
PF in Previous phase
No PF
Start
Goal
Return
Stable
Bidirectional
Context-dependent
Appearing
Disappearing
Pre vs. Learn
Learn vs. Post
Pre vs. Post
Pre
Learn
Post
Z-scored density
3<
0
<-3
Cell characters
Open field
Start, Goal, Return
Stable
Bidirectional
Context
Appearing
Disappearing
Cells
237 place cells in Open field
Stable
Context
38
74
42
34
66
26
10
16
4
29
27
70
39
11
4
25
3
Plotted for "Others" cells
Num. of place fields
All phases
Stable & Context
Others

Fig. S4. Learning-dependent changes in spatial maps. Related to Figure 2. (A) This image is the same as the joint spatial map in Fig. 2D. Place fields (shown in red *) from a single place cell were defined based on spatial firing-rate distributions, and the locations of these place fields were plotted on the X- and Y-axes as black asterisks on a two-dimensional plane. (B) This image is the same as the joint spatial map in Fig. 2E but analyzed separately for all phase pairs. All the place fields from all cells were superimposed. The plots in the thin blue and red regions before the start area represent place fields that were not observed in one phase (No PF) but emerged in the other phase. (C) Density plots of z-scores, constructed from B. In each bin, the density of the dots in B was z-scored based on the average and standard deviation of 1,000 surrogate datasets in which the locations of place fields at the latter phase were randomly shuffled without changing the total number of fields. Higher densities are visible, as shown in Supplementary Fig. 3C and 3D. (D) (top) Hierarchical clustering of all recorded neurons based on their field properties. (bottom) Summary of the field properties of all cells (on, yellow; off, violet). (E) Venn diagram showing the numbers of cells with individual field properties. (F) Same as B but plotted for cells that were classified as “Others” type (n = 154 cells). These cells had place fields in the start area, goal area, and return path, or appearing and disappearing place fields. (G) Comparisons of the number of place fields per place cell across cell types. Each gray dot represents a cell, and the colored lines represent the averages: $^*p < 0.05$, Mann-Whitney U test followed by Bonferroni correction.

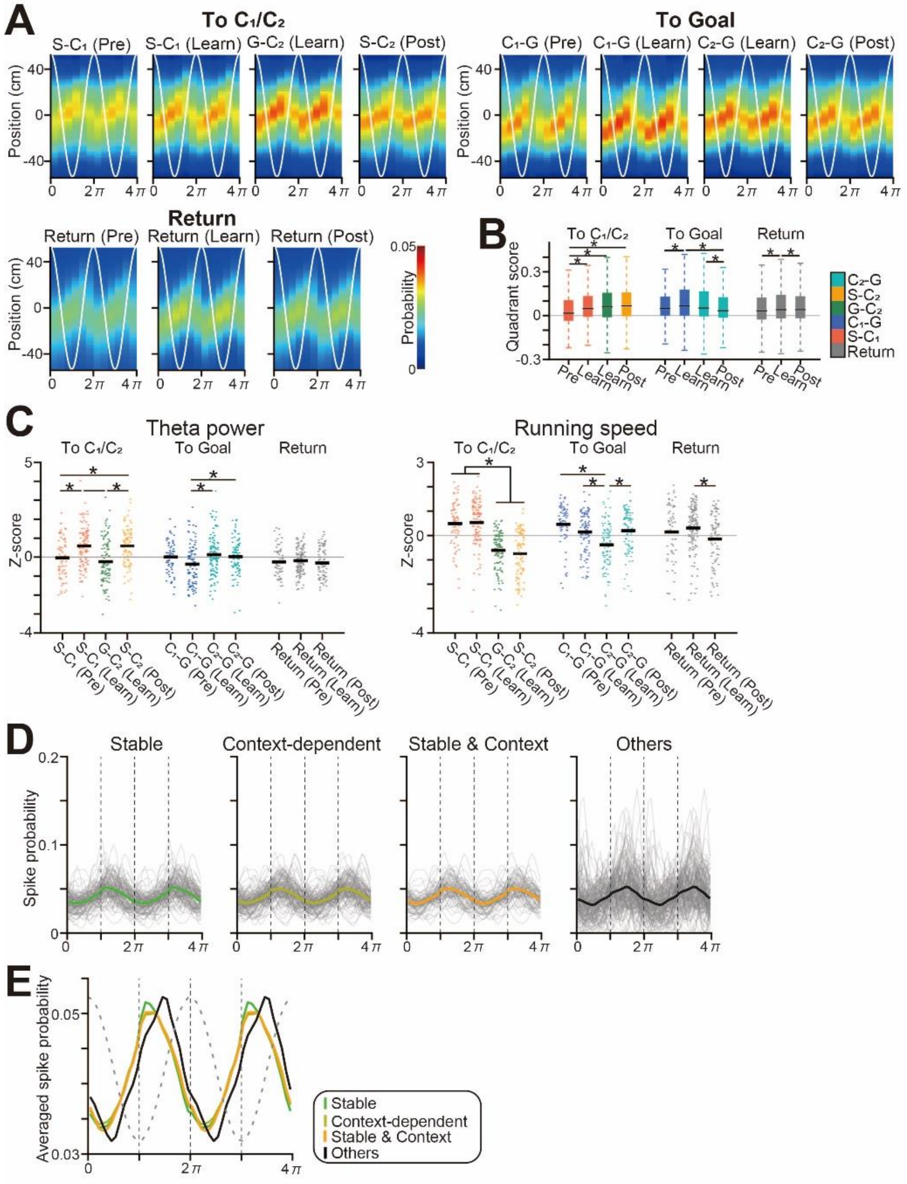

Fig. S5. Learning-dependent and cell-type specific theta sequence. Related to Figure 2. (A) Average posterior probabilities of animals' positions while running on the path indicated above; the x-axis shows the phases of two theta cycles (white line) and the y-axis shows the positions relative to the current animal's location. (B) The bar graphs shown in Fig. 2K are presented as boxplots: $*p < 0.05$, Tukey's test. (C) Theta power and running speed. Each dot represents a trial, and the black lines represent the averages: $*p < 0.05$, Tukey's test. (D) Cells were classified into "Stable", "Context-dependent", "Stable & Context-dependent", and "Others" cells, based on Fig. 2E. The spike probabilities of individual pyramidal cells were plotted against the phase of theta oscillations. Each gray line represents one cell, and the colored lines represent the averages. (E) Superimposition of the average spike probabilities in each cell type corresponding with D.

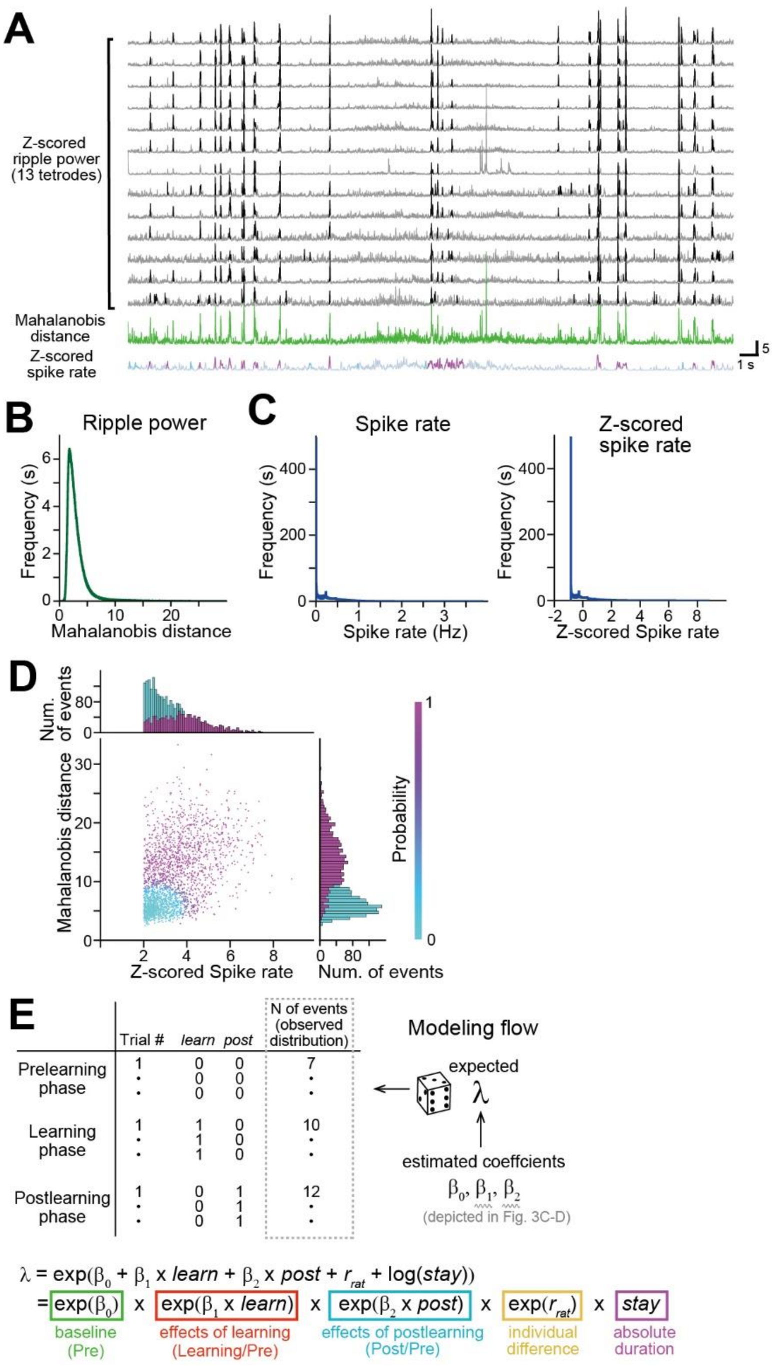

Fig. S6. Detection of SWRs and estimation of synchronous events. Related to Figure 3. (A) From top to bottom are the time changes in z-scored ripple power simultaneously recorded by thirteen tetrodes (upper gray traces), the corresponding Mahalanobis distance computed from the thirteen 150–250 Hz bandpass-filtered LFP traces as overall ripple power (green trace), and the z-scored spike rates of all recorded hippocampal cells (bin = 1 ms; sigma = 15 ms for Gaussian filter). Ripple power traces were computed from the 150–250 Hz bandpass-filtered LFP traces by the Hilbert transform (sigma = 4 ms for Gaussian filter), and the times at which ripples were detected in individual electrodes are colored in black. (B, C) The frequency distributions of the Mahalanobis distance (B) and the average spike rates of hippocampal cells: (C, left) (bin

= 1 ms). The average spike rates are z-scored in the rightmost panel. (D) Relationship between the Mahalanobis distance and z-scored spike rates. Synchronous events were defined when the dots were included in the magenta cluster. (E) Schematic illustration of computation of changes in the frequency (or the number) of synchronous events. Synchronous events were assumed to emerge according to Poisson statistics (indicated by dice) with an expected value, $\boldsymbol{\lambda}$, representing the number of synchronous events per second (or trial). The dummy variables $\boldsymbol{learn}$ and $\boldsymbol{post}$ are 1 in each corresponding phase, whereas they are 0 in the other phases. In the bottom panel, the equation in the Methods was expanded into the second formula, demonstrating that $\boldsymbol{\lambda}$ is the multiplication of a series of distributions defined by the estimated coefficients, $\boldsymbol{\beta_0}, \boldsymbol{\beta_1}, \mathbf{and}\ \boldsymbol{\beta_2}$, which were considered as the baseline (prelearning phase), the effects of learning and postlearning phases relative to the prelearning phase, respectively. In Figure 3C and 3D, the distributions were depicted as $\mathbf{100} \times [\mathbf{exp}(\boldsymbol{\beta_i}) - \mathbf{1}]$, where *i* = 1 or 2, representing the learning or postlearning phases, respectively. A term, $\boldsymbol{r_{rat}}$, was included to compensate for the fluctuations and the noise of baseline datasets across individual rats, which took any normal distributions with a mean of 0 and a variance ranging from 0 to 10^4 (determined for convenience). A term, *stay*, was the duration to define synchronous events, which was included when the number of synchronous events was counted from variable duration, whereas it was set to be 1 when the duration was not needed to be considered (Supplementary Fig. 7D).

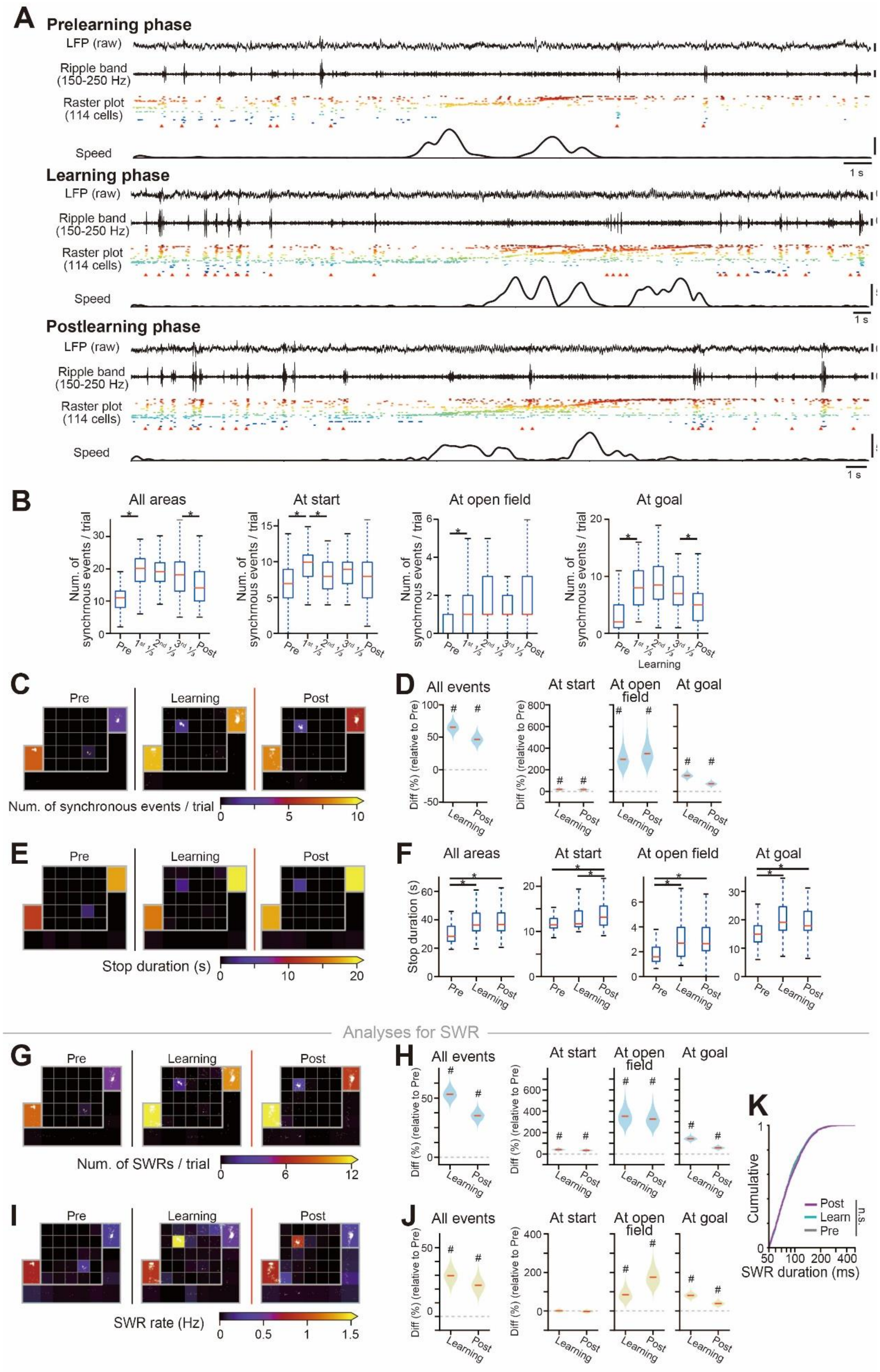
A Prelearning phase
LFP (raw)
Ripple band (150-250 Hz)
Raster plot (114 cells)
Speed
1 s
Learning phase
Postlearning phase
B All areas
At start
At open field
At goal
Num. of synchronous events / trial
Pre
Post
Learning
C Pre
Learning
Post
Num. of synchronous events / trial
D All events
Diff (%) (relative to Pre)
E Stop duration (s)
F All areas
Stop duration (s)
Analyses for SWR
G Num. of SWRs / trial
H All events
I SWR rate (Hz)
J All events
K Cumulative
Post
Learn
Pre
n.s.
SWR duration (ms)

Fig. S7. Learning-dependent changes in the numbers of synchronous events and SWRs. Related to Figure 2. (A) From top to bottom are the original LFP, ripple band-filtered (150–250 Hz) LFP traces, a raster plot of the spike patterns of 114 neurons where the bottom arrowheads indicate synchronous events, and rat running speed during a single trial of each phase. Compared with the learning and postlearning phases, smaller numbers of synchronous events and SWRs are visible in the prelearning phase. (B) The numbers of synchronous events detected in each learning (sub)phase. $^*p < 0.05$, Mann-Whitney U test followed by Bonferroni correction. (C) Pseudocolor maps of the average number of synchronous events during stop periods in each trial. The locations of synchronous events are indicated by superimposed white dots. (D) Same as Fig. 2C and 2D but analyzed for the absolute number of synchronous events relative to those in the prelearning phase. A pound sign (#) indicates no overlap between 0 and the 95% credible intervals computed from the posterior probability distribution by MCMC. (E) Pseudocolor maps of the average stop duration (moving speed less than 5 cm/s) in each trial. (F) Comparisons of stop duration among the prelearning, learning, and postlearning phases. $^*p < 0.05$, Mann-Whitney U test followed by Bonferroni correction. (G, H) Same as C and D but analyzed for the number of SWRs. (I, J) Same as C and D but analyzed for the frequency of SWRs. (K) Cumulative distributions of SWR duration. No significant differences were found across the phases: $p > 0.05$, Mann-Whitney U test followed by Bonferroni correction.

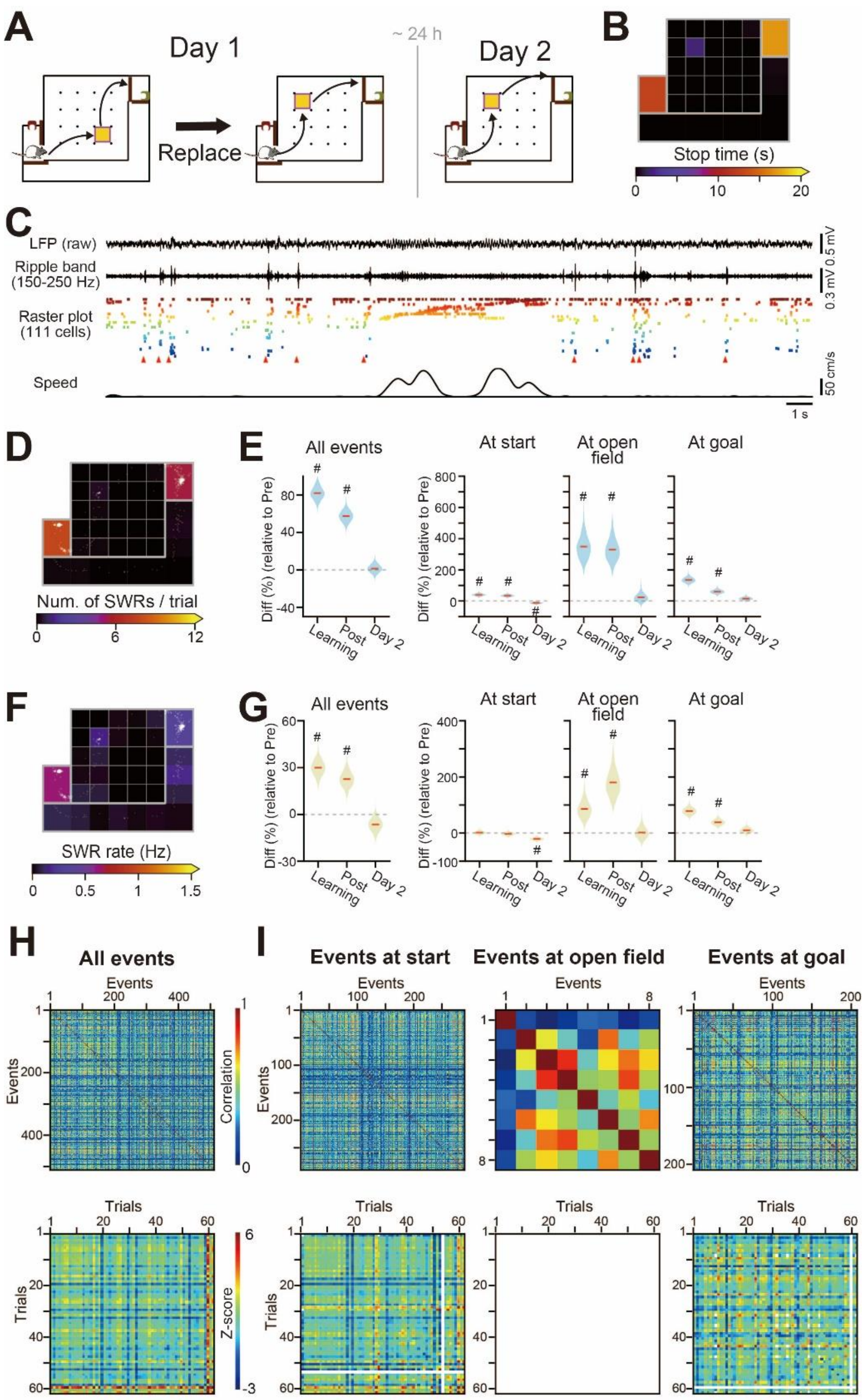

Fig. S8. No increases in SWRs were observed under any learning conditions. Related to Figure 3. (A) On the next day after the rat fully learned a checkpoint (C_2), the same recording commenced without a change to the reward point (day 2), meaning that no further learning was induced. In this figure, all the analyses were performed from the data collected on day 2 unless otherwise specified. (B) A pseudocolor map of the average stop duration (moving speed less than 5 cm/s) in each trial on day 2. (C) From

top to bottom are the original LFP, ripple band-filtered (150–250 Hz) LFP traces, a raster plot of spike patterns where the bottom arrowheads indicate synchronous events, and rat running speed from in a representative trial on day 2. Note that there are fewer synchronous events and SWRs compared with those in Fig. 1H. (D, F) Pseudocolor maps showing the number (D) and frequency (F) of SWRs per trial using the same color scales as in Supplementary Fig. 7G and 7I. The locations of synchronous events are indicated by superimposed white dots. (E, G) The percentage of changes in the number and frequency of SWRs during the learning and postlearning phases on day 2 compared to the prelearning phase. Data from the learning and postlearning phases are similar to those shown in Supplementary Fig. 7H and 7J for comparison. On day 2, no significant increases in SWRs were observed compared with the prelearning phase. A pound sign (#) indicates no overlap between 0 and the 95% credible intervals computed from posterior probability distribution by MCMC (*n* = 4–5 rats). (H) (top) An event-to-event correlation matrix of synchronous events on day 2. Detailed explanations are provided in Supplementary Figure 9. (bottom) A trial-to-trial correlation matrix constructed from the event-to-event matrix in which the correlation coefficients are shown as z-scores computed from 1,000 surrogate datasets, showing no prominent changes in coefficients throughout the experiment, compared with those in Supplementary Fig. 9B-D. (I) Similar to H but separately analyzed for the individual areas. Trials with fewer than 3 synchronous events were not analyzed and are shown in white.

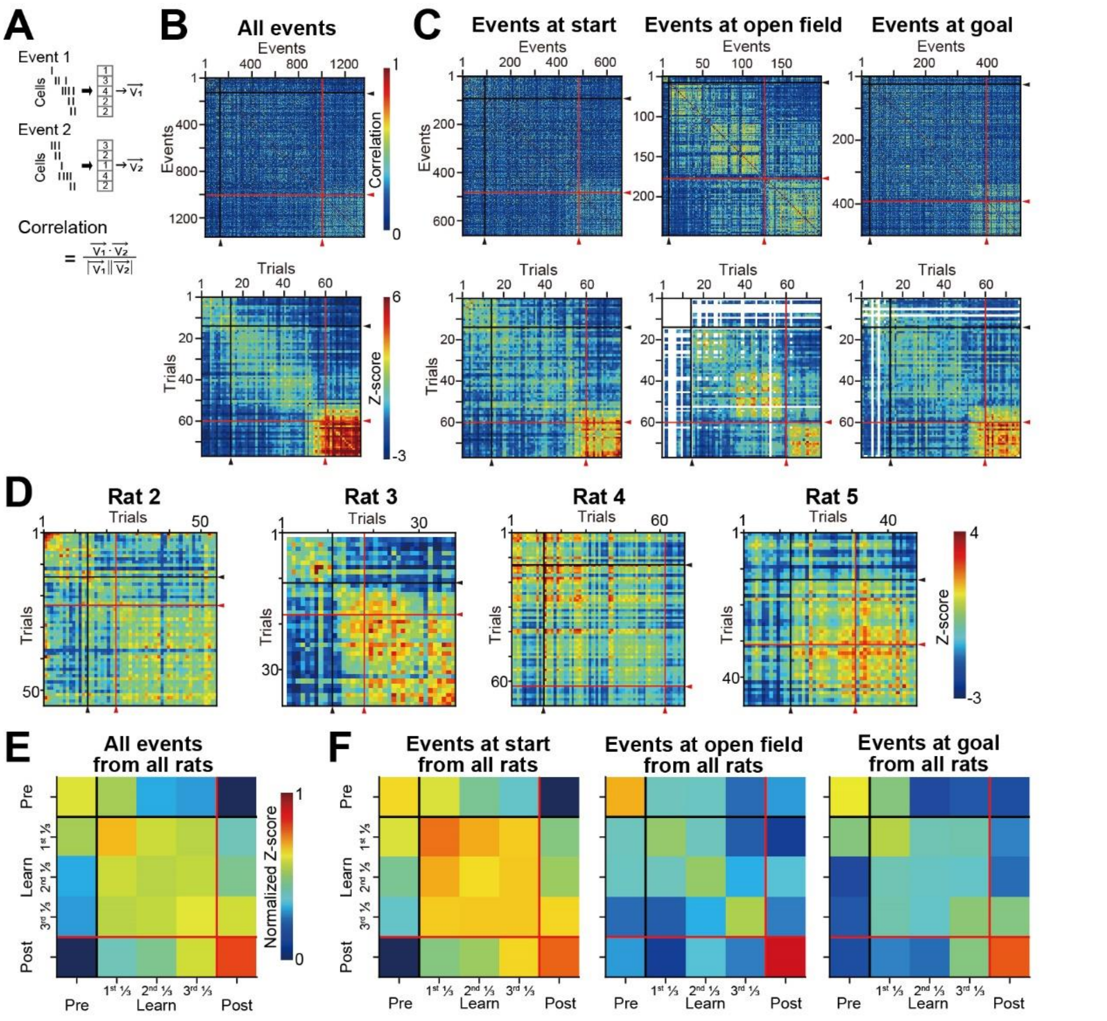

Fig. S9. Correlation of spike patterns in synchronous events. Related to Figure 3. (A) A population vector was constructed from the spike counts of all cells for each synchronous event. Correlation coefficients were computed from all pairs of population vectors and used to construct an event-to-event correlation matrix. (B) (top) An event-to-event correlation matrix of all synchronous events observed at all areas for one rat (rat 1). The black and red lines indicate the reward replacement from C_1 to C_2 and the learning point, respectively. (bottom) A trial-to-trial correlation matrix constructed from the event-to-event matrix by calculating the average of all the correlation coefficients included in each trial pair. Correlation coefficients are shown as z-scores based on 1,000 surrogate datasets. (C) Similar to B but separately analyzed for the individual areas. Trials with fewer than 3 synchronous events were not analyzed and are shown in white. Higher correlations are visible within each learning and postlearning phase compared with those within the prelearning phase. (D) Trial-to-trial correlation matrices in the other individual rats (rats 2–5). (E, F) Similar to the trial-to-trial correlation matrix, a phase-to-phase z-scored correlation matrix was constructed from the event-to-event matrix. A z-scored event-to-event matrix was first normalized so that the minimum and maximum values were 0 and 1, respectively, in each rat; then, the normalized matrices were averaged across all rats. These correlation matrices show that synchronous event patterns undergo continuous changes throughout the learning phase.

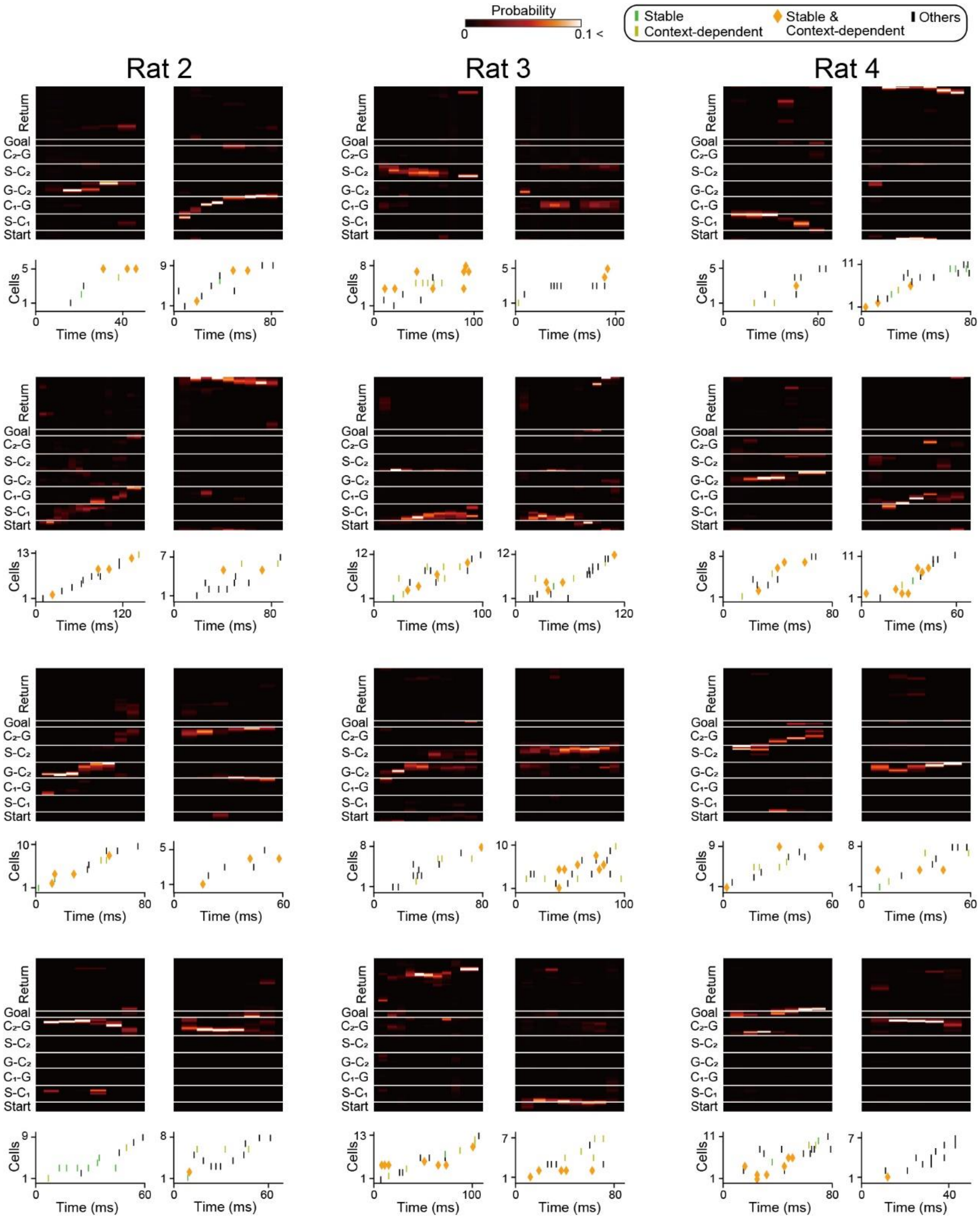

Fig. S10. Bayesian decoding of animal trajectories from individual synchronous events (from Rat 2-4). Related to Figure 3. For each rat, eight synchronous events are presented. Each panel shows posterior probabilities of position estimates for each synchronous event (top) and the corresponding raster plots of place cell spikes used for decoding, labeled based on cell type (bottom). Cell numbers were sorted according to spike timing within each event.

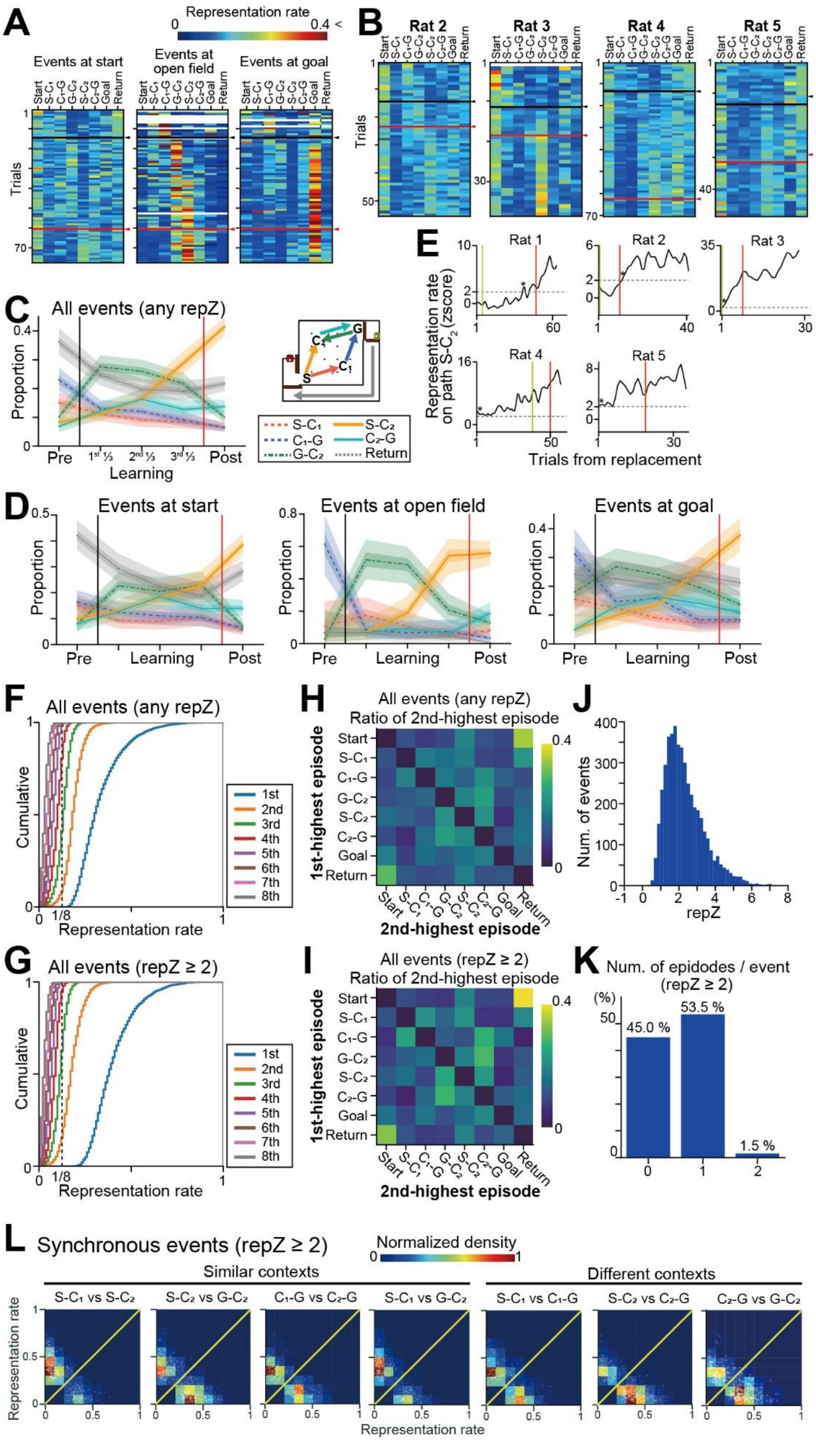

A
Representation rate
0
0.4 <
Events at start
Events at open field
Events at goal
Trials
B
Rat 2
Rat 3
Rat 4
Rat 5
Start
S-C₁
C₁-G
G-C₂
S-C₂
C₂-G
Goal
Return
E
Rat 1
Representation rate on path S-C₂ (zscore)
Trials from replacement
C
All events (any repZ)
Proportion
Pre
Post
Learning
S-C₁
S-C₂
C₁-G
C₂-G
G-C₂
Return
D
Events at start
Events at open field
Events at goal
F
All events (any repZ)
Cumulative
Representation rate
1st
2nd
3rd
4th
5th
6th
7th
8th
H
All events (any repZ)
Ratio of 2nd-highest episode
1st-highest episode
2nd-highest episode
J
Num. of events
repZ
G
All events (repZ ≥ 2)
I
All events (repZ ≥ 2)
K
Num. of epidodes / event (repZ ≥ 2)
(%)
45.0 %
53.5 %
1.5 %
L
Synchronous events (repZ ≥ 2)
Normalized density
Similar contexts
Different contexts
S-C₁ vs S-C₂
S-C₂ vs G-C₂
C₁-G vs C₂-G
S-C₁ vs G-C₂
S-C₁ vs C₁-G
S-C₂ vs C₂-G
C₂-G vs G-C₂
Representation rate

Fig. S11. Represented paths by synchronous events. Related to Figure 3. (A) Color-coded matrices showing changes in representation rates for each path by synchronous events, separately analyzed for individual areas (the same rat shown in Fig. 3F). The black and red lines indicate the reward replacement from C_1 to C_2 and the learning point, respectively. (B) Same as Fig. 3F but for the other individual rats (rats 2–5). (C) Same as Fig. 3G but for learning-related changes in the percentage of all synchronous events, irrespective of their *repZ*, representing individual paths. The thick and thin shaded areas indicate the 50% and 95% credible intervals, respectively. Similarly, pronounced increases in S-C_2 are visible in the latter learning phases. (D) Same as C but separately plotted for the individual areas. (E) Changes in representation rates of path S-C_2 during the learning phase in each rat. Representation rates of path S-C_2 were zscored based on an average and a standard deviation computed from those in the prelearning phase. The yellow and red vertical lines indicate the time when the rat first passed path S-C_2 (for more detail, see Supplementary Figure 2C) and the learning point, respectively. The onset of an increase in the representation rates (when the zscored rate first exceeded 2) is indicated by the asterisk. (F, G) To analyze how the representation rates (*reprates*) of each synchronous event were biased for particular paths, z-scored representation rates (*repZ*) for all paths were computed for each synchronous event, and these *reprates* were ranked from highest (1st) to lowest (8th). For each rank, cumulative distributions were depicted from the *reprates* of all synchronous events (F) and synchronous events with *repZ* $\geq$ 2. (H, I) The probability of detecting synchronous events with the 1st and 2nd *reprate* at paths (or areas) indicated by the y-axis and x-axis, respectively. In I, a higher probability is visible in the comparison of neighboring paths (i.e., S-C_1 versus C_1-G and Start versus Return), implying the presence of joint replays of multiple episodes that are close in time and space. (J) The distribution of the 1st *repZ* of all synchronous events. Synchronous events whose *repZ* $\geq$ 2 were considered to represent specific paths. (K) The number of represented paths identified from single synchronous events. (L) Comparison of representation rates decoded from synchronous events. For each synchronous event with *repZ* $\geq$ 2, representation rates of two given path segments with similar (left four panels) and different (right three panels) contexts were compared (similar to Supplementary Figure 3G). Each white dot indicates each synchronous event, superimposed on a pseudocolor matrix of the normalized densities of all plots. In all comparisons, the majority of dots was plotted on the regions with a *reprate* close to 0.5 in one path segment and a *reprate* close to 0 in the other segment, not plotted around the diagonal lines (yellow lines), suggesting that the majority of synchronous events primarily represented one path segment.

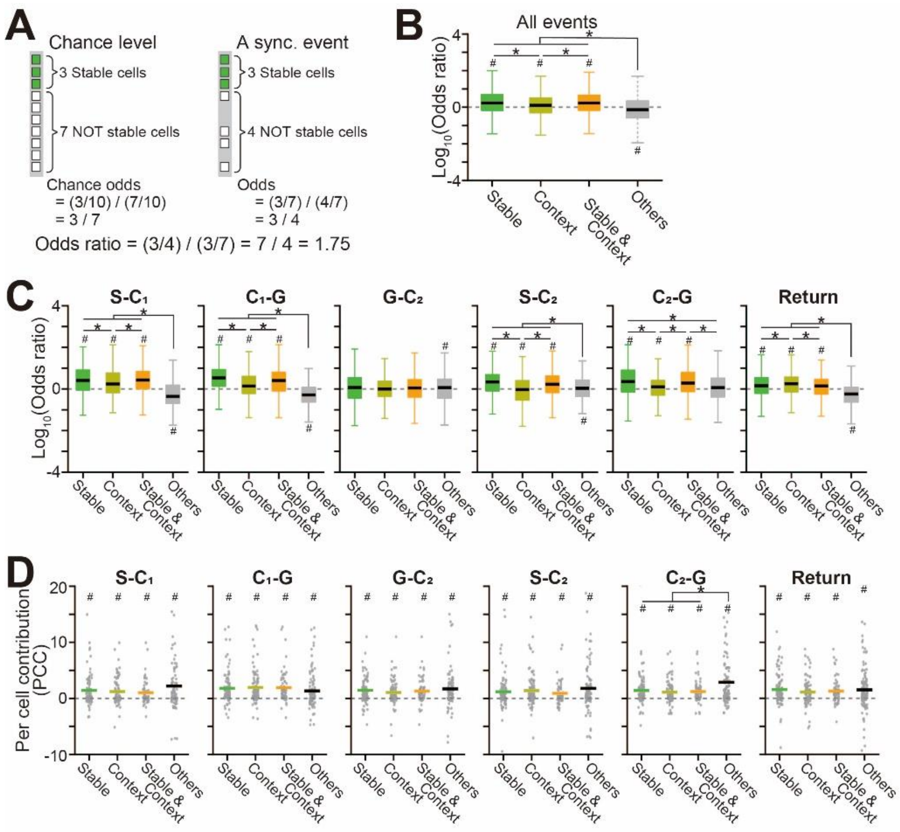

Fig. S12. Cell participation in synchronous events and per cell contribution to replay events. Related to Figure 4. (A) Schematic illustration of computing the odds ratios for a cell type. This illustration includes a total of 10 place cells with 3 stable cells (green boxes) and 7 other cells (white boxes). An example synchronous event includes 3 and 4 active cells. For this synchronous event, the odds were computed as the ratio of the percentage of active stable cells to total active cells (3/7) to the percentage of active other cells to total active cells (4/7). The odds at a chance level were computed as the ratio of the percentage of total stable cells to total cells (3/10) to the percentage of total other cells to total cells (7/10). To compute a chance level for each path, the active place cells in the path were analyzed. Finally, the odds ratio was computed as a ratio of the two odds as shown below. (B) Comparison of odds ratios across cell types: #$p < 0.05$, one-sample t-test versus 0; *$p < 0.05$, Tukey's test. (C) Same as B but separately analyzed for the individual paths. (D) Per cell contribution of individual cells to replay events, separately analyzed for the individual represented paths: #$p < 0.05$, one-sample t-test versus 0; *$p < 0.05$, Tukey's test.

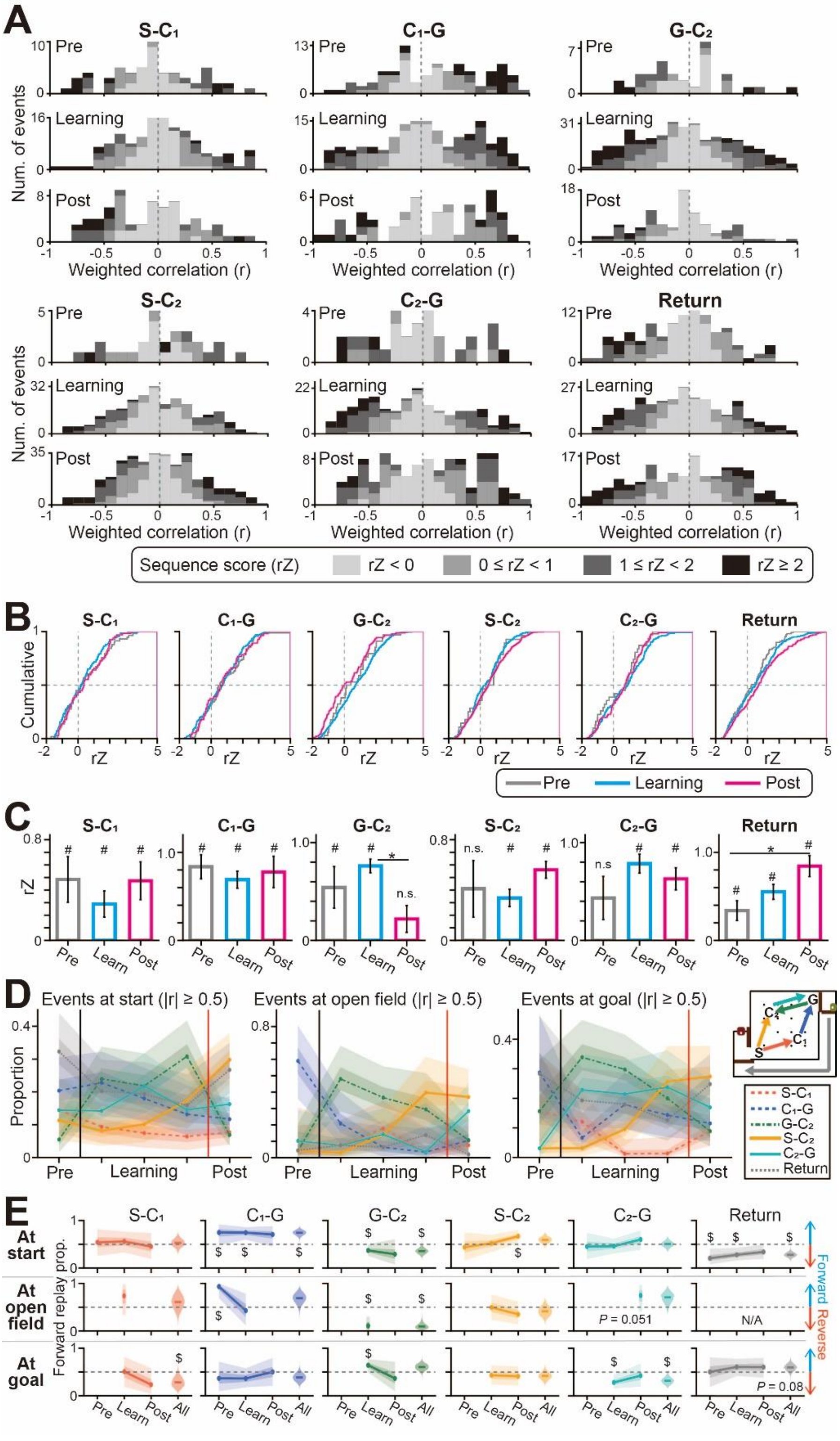

Fig. S13. Sequential events representing individual paths. Related to Figure 4. (A) Distributions of the weighted correlations (*r*) of synchronous events. The positive and negative *r* values represent forward and reverse directions, respectively. Each event is labeled in color depending on its sequence score (*rZ*). Synchronous events with an |*r*| above 0.5 were considered to be sequential events. (B) Cumulative distributions of the z-

scored sequence scores (*rZ*) of synchronous events, separately analyzed for the individual represented paths. (C) The same data shown in B were averaged: #$p < 0.05$, one-sample t-test versus 0; *$p < 0.05$, Tukey's test. (D) Same as Fig. 3F but separately analyzed for replay events in the individual areas. The thick and thin shaded areas indicate the 50% and 95% credible intervals, respectively. (E) The directionality of replay events separately analyzed for individual paths. The thick and thin shaded areas indicate the 50% and 95% credible intervals, respectively. A dollar ($) symbol indicates that the overlap in the distribution probability between 0.5 and the posterior probability distribution was less than 5%. Remarkably, the results showed that (1) replay events representing the return path observed at the start and goal areas were biased toward the reverse and forward directions, respectively, and (2) replay events representing paths G-C_2 and C_2-G observed in the open field (the majority of which emerged at C_2 as shown in Fig. 2B) were biased toward the reverse and forward directions, respectively. These results demonstrate that forward and reverse replays preferentially emerge when their represented paths correspond with an animal's behavioral episodes in the immediate future and past, respectively. Together with the results showing that reverse replays were more dominant than were forward replays in the open field (the majority of which emerged at C_2) during the learning phase (Fig. 3D), path G-C_2 is a main episode that is preferentially represented by reverse replays during the learning.

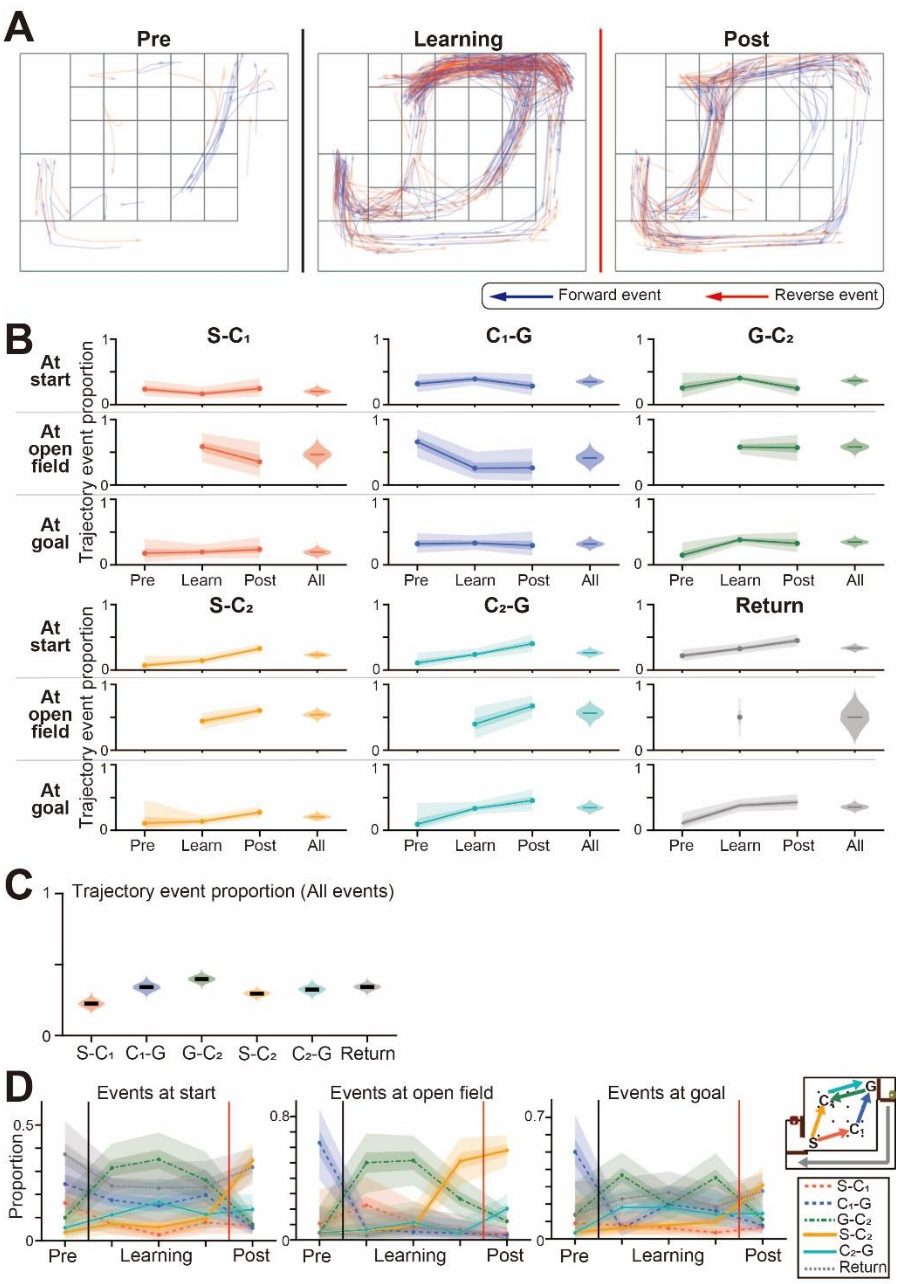

Fig. S14. Representation by trajectory events. Related to Figure 4. (A) Superimposition of the decoded trajectories from all trajectory events in a rat. The forward and reverse trajectory events are shown as blue and red lines, respectively. (B) The percentage of synchronous events to be assigned as trajectory events. The thick and thin shaded areas indicate the 50% and 95% credible intervals, respectively. (C) The same data shown in B were superimposed for comparison across paths. (D) The percentage of represented paths by trajectory events, separately analyzed for the individual areas. The thick and thin shaded areas indicate the 50% and 95% credible intervals, respectively.

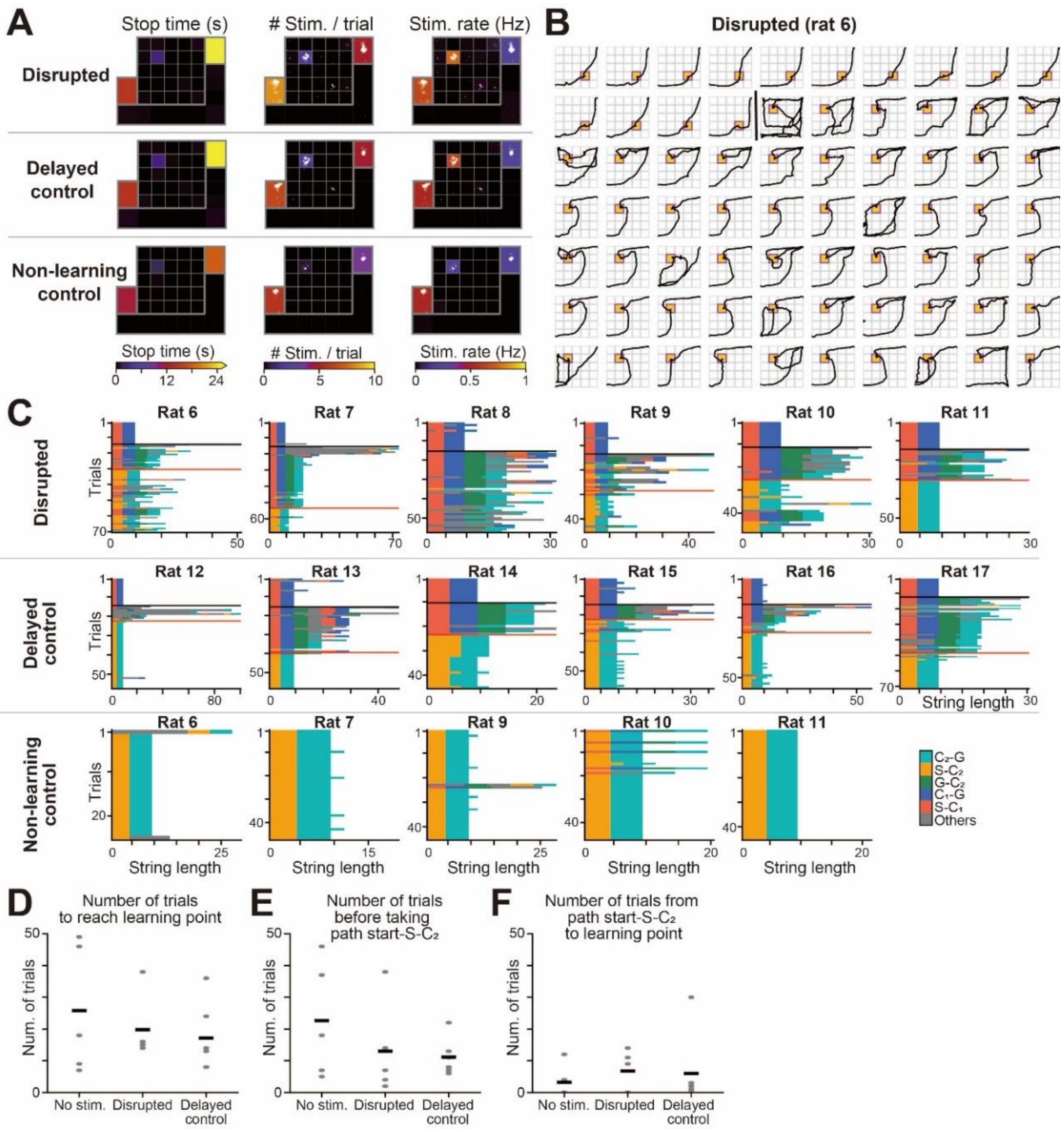

Fig. S15. Effects of SWR inhibition on behavioral performance. Related to Figure 5. (A) Pseudocolor maps of the average stop duration (moving speed less than 5 cm/s) and the number and frequency of feedback electrical stimulation applied per trial. Stimuli locations are indicated by superimposed white dots. (B) Representative running trajectories observed in a single rat with disrupted SWRs. The black vertical line indicates reward replacement. (C) Changes in string lengths for the other individual rats in the disrupted SWR, delayed control, and nonlearning control groups. The black and red horizontal lines indicate the reward replacement and the learning point, respectively. The data are colored depending on the paths taken by the rats. (D) The number of trials to reach the learning point in each group. Each dot represents a rat, and the black lines represent the averages. *P* > 0.05, Tukey's HSD post hoc test. (E) The number of trials until the rats first took the path start-S-C_2. *P* > 0.05, Tukey's HSD post hoc test. (F) The number of trials from when rats first took the path start-S-C_2 to the learning point. *P* > 0.05, Tukey's HSD post hoc test.

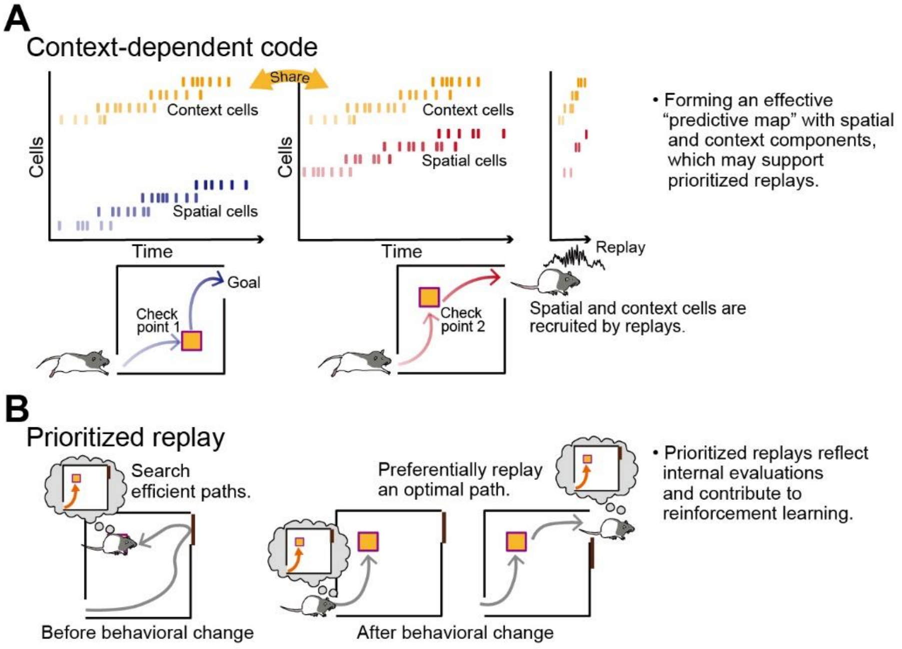

Fig. S16. Schematic illustrations suggested from this study. (A) Context-dependent codes on a predictive map. Context-dependent cells, which encode task contexts (e.g. toward check points), are shared across learning phases and are recruited by hippocampal replays. The brain circuits form an effective predictive map with both spatial and context components, which may support memory processes and prioritized replays. (B) Prioritized replays on a predictive map. Hippocampal circuits preferentially replay salient episodes based on agent's internal evaluations in a model in the brain. Such prioritized replays likely contribute to efficient learning.

Movie S1 (separate file). Bayesian decoding of animal trajectories from synchronous events. The posterior probabilities of position estimations are represented by a hot scale. The green circles represent the animals' current locations. The movie shows that decoded trajectories transiently emerge, and that synchronous events corresponding to forward and reverse replays are enriched during the learning and postlearning phases. The movie speed is equivalent to and two times higher than an actual speed during the emergence of synchronous events and the other periods, respectively.

Movie S2 (separate file). Learning-dependent changes in the represented paths by synchronous events. Similar to Fig. 2f, the averaged representation rates of synchronous events with repZ ≥ 2 for the individual paths are visualized by a pseudocolor scale in each trial for a rat. The white line represents the actual path taken by the rat in each trial.

SI references

1. S. P. Jadhav, C. Kemere, P. W. German, L. M. Frank, Awake hippocampal sharp-wave ripples support spatial memory. *Science* **336**, 1454-1458 (2012).
2. A. D. Redish, MClust 3.5, Free-Ware Spike Sorting (University of Minnesota, Minneapolis). Available at http://redishlab.neuroscience.umn.edu/MClust/MClust.html. (2009).
3. N. Schmitzer-Torbert, J. Jackson, D. Henze, K. Harris, A. D. Redish, Quantitative measures of cluster quality for use in extracellular recordings. *Neuroscience* **131**, 1-11 (2005).
4. A. D. Grosmark, G. Buzsaki, Diversity in neural firing dynamics supports both rigid and learned hippocampal sequences. *Science* **351**, 1440-1443 (2016).
5. B. E. Pfeiffer, D. J. Foster, Hippocampal place-cell sequences depict future paths to remembered goals. *Nature* **497**, 74-79 (2013).
6. T. Feng, D. Silva, D. J. Foster, Dissociation between the experience-dependent development of hippocampal theta sequences and single-trial phase precession. *The Journal of neuroscience : the official journal of the Society for Neuroscience* **35**, 4890-4902 (2015).
7. R. E. Ambrose, B. E. Pfeiffer, D. J. Foster, Reverse Replay of Hippocampal Place Cells Is Uniquely Modulated by Changing Reward. *Neuron* **91**, 1124-1136 (2016).
8. X. Wu, D. J. Foster, Hippocampal replay captures the unique topological structure of a novel environment. *The Journal of neuroscience : the official journal of the Society for Neuroscience* **34**, 6459-6469 (2014).
9. A. A. Carey, Y. Tanaka, M. A. A. van der Meer, Reward revaluation biases hippocampal replay content away from the preferred outcome. *Nature neuroscience* **22**, 1450-1459 (2019).
10. A. Gelman *et al.*, *Bayesian Data Analysis, Third Edition (CRC Press, 2013)* (2013).
